# Quantitative assessment of visual designs for communicating patient-reported outcomes in breast cancer care to patients

**DOI:** 10.1186/s41687-025-00984-0

**Published:** 2025-12-20

**Authors:** Lea Doppelbauer, Anna Maria Hage, Maria Margarete Karsten, Pimrapat Gebert, Anna Tatzber, Laura Hatzler, Jasper Brands, Rosanne Andriessen, Therese Pross

**Affiliations:** 1 Charité Universitätsmedizin Berlin, Department of Gynecology with Breast Center, Charité—Universitätsmedizin Berlin, Corporate Member of Freie Universität Berlin and Humboldt Universität zu Berlin, 10117 Berlin, Germany; 2 Charité Universitätsmedizin Berlin, Institute of Biometry and Clinical Epidemiology, Charité—Universitätsmedizin Berlin, Corporate Member of Freie Universität Berlin and Humboldt Universität zu Berlin, 10117 Berlin, Germany; 3 Charité Universitätsmedizin Berlin, Department of Urology, Institute of Sexology and Sexual Medicine, Charité—Universitätsmedizin Berlin, Corporate Member of Freie Universität Berlin and Humboldt Universität zu Berlin, 10117 Berlin, Germany; 4 Panton bv, Muntengang 1, Deventer, 7411 GD Netherlands

**Keywords:** Patient-reported outcomes, Breast cancer, Data visualization, Qualitative data, Design concept evaluation, Health communication, Patient-centered care, Health literacy

## Abstract

**Background:**

Patient-reported outcomes (PROs) are increasingly recognized as valuable data to enhance communication and support shared decision-making in cancer care. Despite evidence of their positive effects, few concrete approaches exist for the design and presentation of PRO visualizations. Even in high-incidence cancers such as breast cancer, limited knowledge is available on how patients perceive and interpret graphical displays of PRO results. This study evaluated different visualization formats of PRO data, that had been co-designed with patients and healthcare providers within the PRO B study, a large randomized controlled trial investigating alert-based PRO monitoring in patients with metastatic breast cancer.

**Methodology:**

After completion of the PRO B study, patients from both intervention and control group received a paper-based questionnaire together with their individual PRO data report featuring visualizations developed in a preceding design study. Survey items assessed comprehensibility, usefulness, and preferences regarding specific visualization features using Likert scales. Analyses included Mann-Whitney U tests for Likert scale answers, Pearson’s Chi-squared tests for categorical variables and two-sided independent-samples t-tests for numeric variables.

**Results:**

We collected feedback from 276 participants of the PRO B study. The findings demonstrate a strong overall endorsement on the clarity, emotional appropriateness and usefulness of the suggested graphical displays. Results highlight their potential for enhancing patient empowerment through improved self-awareness and better patient-provider communication.

**Conclusions:**

The positive feedback from PRO B study participants validate the suggested design concepts of PRO data and underscores their valuable role in making these outcomes more accessible, meaningful, and actionable for patients. These data provide the base for developing more flexible, interactive digital tools for routine care.

**Trial registration:**

DRKS00024015 Registered 15.02.2021 https://drks.de/search/de/trial/DRKS00024015/details.

**Supplementary Information:**

The online version contains supplementary material available at 10.1186/s41687-025-00984-0.

## Abstract (plain language summary)

Patient-reported outcomes (PROs) refer to information that patients provide about their symptoms, daily functioning, and quality of life, often collected through standardized questionnaires. These questionnaires collect patient-reported outcomes (PROs) - information that reflects how patients perceive their health and well-being. These insights can give doctors information that medical tests alone cannot provide. PRO data can improve communication and support shared decision-making in cancer care. However, the way PRO results are usually presented in charts and graphs can be confusing and sometimes overwhelming for patients.

In this study, we tested new types of visualizations for PRO data that were co-designed with patients and healthcare professionals. We asked 276 women with metastatic breast cancer to review their own results displayed in these formats and to share their views. Patients evaluated how clear, useful, and emotionally acceptable they found the displays.

The majority reported that the new visualizations were easy to understand, supportive for self-reflection, and helpful for conversations with doctors. Our findings suggest that carefully designed, flexible visualizations can make PRO data more accessible and meaningful, thereby promoting more patient-centered cancer care.

**Supplementary Information:**

The online version contains supplementary material available at 10.1186/s41687-025-00984-0.

## Background

Patient-reported outcomes (PROs i.e. the information directly reported by patients about their health status) are collected using patient-reported outcome measures **(**PROMs**)** and are a cornerstone of modern oncology, offering structured insights into patients’ symptoms, functional status, and quality of life. When integrated into routine clinical care, PRO data can improve symptom management, enhance communication between patients and providers, and even contribute to improved survival and reduced hospitalizations [1–3]. However, the effectiveness of PRO-based monitoring systems in practice hinges not only on routinely collected data [4, 5], but also on how this information is communicated and understood by its intended users – patients and healthcare providers alike [5].

Due to a lack of empirically supported methods for displaying PRO data, they are often presented through raw scores or complex tables that are difficult to interpret, particularly for patients with lower health literacy [6–9]. However, to support shared decision-making and improve usability, PRO data must be presented in a format that is both intuitive and visually accessible for all users [10].

Recent work within the multicentric, randomized PRO B study [11], which evaluated an alert-based symptom monitoring system for patients with metastatic breast cancer, highlighted the need for more intuitive and emotionally sensitive visualization of PRO data. Thus, in a preceding qualitative sub-study of the PRO B trial, new visualization concepts for PRO data were developed using healthcare service design principles and participatory design, iteratively refining graphical visualizations through interviews with patients and providers [12]. In qualitative interviews, aspects like preferences regarding visual elements (e.g., color schemes, labeling, alert design), perceived interpretability, emotional impact, and the possible benefit of PRO visualizations in breast cancer treatment were addressed. These efforts aimed to enhance clarity, usability, and emotional appropriateness of PRO data displays, particularly in the context of long-term disease monitoring.

While qualitative feedback suggested strong acceptance and perceived value of the proposed visualization concepts, the generalizability and broader applicability of these findings remained to be tested. Furthermore, systematic evaluation of user preferences, perceived usefulness, and potential for routine implementation requires robust quantitative data from a larger, more diverse sample of users.

To address this gap, the present study aimed to evaluate the user-centered PRO visualizations developed during the PRO B study using a standardized quantitative survey administered to patients and healthcare professionals at the end of the PRO B study. Specifically, we assessed the comprehensibility of the designed visualizations, the need for additional information and content in the visualizations and their practical utility in clinical care. In doing so, this study provides an empirical foundation for future implementation of optimized PRO visualizations in oncology and offers concrete guidance for the design of patient-centered health data displays.

## Methods

### The PRO B trial

This sub-study was embedded in the PRO B trial, a multicenter randomized controlled study evaluating the impact of alert-based PRO monitoring in patients with metastatic breast cancer across 52 German breast cancer centers [11, 13]. Conducted between May 2021 and February 2024, PRO B enrolled 924 patients randomized to an intervention or control group. In addition to routine care, intervention group participants completed weekly PRO-based symptom assessments via smartphone app. PRO scores were accessible in real time to the patients’ care teams through a web-based reporting platform that included a basic version of PRO visualization, preceding the advanced formats evaluated in this study. If scores worsened, the system automatically generated an email alert prompting the care team to contact the patient within 48 h to assess health status and provide support as needed. Patients could not view their own PRO results and were unaware if their responses had triggered an alert. Control group participants completed PRO surveys every three months without alert-based monitoring.

### Study design and setting

Embedded in the PRO B trial, a qualitative design project was conducted to develop and co-design new visualization prototypes of PRO data with patients and healthcare providers, by considering existing literature on health data visualization. The details of this sub-study are described in a companion manuscript [12]. Final design decisions are described in 2.5 and Table 1. The present study is a quantitative follow-up to this qualitative concept study, aimed to systematically assess the acceptance, comprehensibility and interpretability, as well as usability of these new design concept for PRO data through a structured survey among patients participating in the PRO B study. Ethical approval was obtained.

### Survey development

The survey was designed collaboratively by the PRO B study team (including clinical experts, researchers and a study nurse), two patient advocates, members of the German Cancer Society (Deutsche Krebsgesellschaft, DKG) as well as the healthcare service design team involved in the preceding qualitative design project. It was based on key findings from literature review and qualitative interviews of the design project and included Likert-scale items assessing specific visualization features (e.g., labeling, color schemes, alert representation, reference data) as well as global questions on perceived clarity, usefulness, and emotional response. The survey was part of a larger questionnaire evaluating the PRO B study in general, including sociodemographic questions and items capturing participants’ experience with PRO monitoring. A pilot version of the survey was pretested for clarity and face validity with a small group of patients (*n* = 5) and healthcare professionals (*n* = 3) not involved in the earlier design study. Minor wording adjustments were made based on their feedback.

### Participants and survey distribution

At the end of the PRO B study, participants received a paper-based survey along with a personalized monitoring report displaying their PRO scores from the previous 12 months, visually designed according to the findings of the preceding design concept project. This ensured that the survey responses directly reflected the evaluation of the newly designed visual elements and their potential implications for PRO reporting. In May 2024, surveys and reports were distributed to all patients of the main trial who did not fulfill the following exclusion criteria: death, retracted informed consent during the PRO B trial, no answered PRO questionnaires during the last 6 months. Consequently, all eligible patients had previously completed regular PRO assessments during the PRO B study, weekly in the intervention group and every three months in the control group, with these differing assessment intervals reflected in the visualizations.

Since the coordinating study center only had access to pseudonymized IDs of study participants, surveys and reports were sent to the respective study sites first, which maintained the identifying information. From there, the reports were distributed to the individual patients. The survey (Online Resource 1) and example PRO reports (intervention group: Online Resource 2, control group: Online Resource 3) are provided online.

### Design decisions from preceding qualitative design concept study

**Fig. 1:**
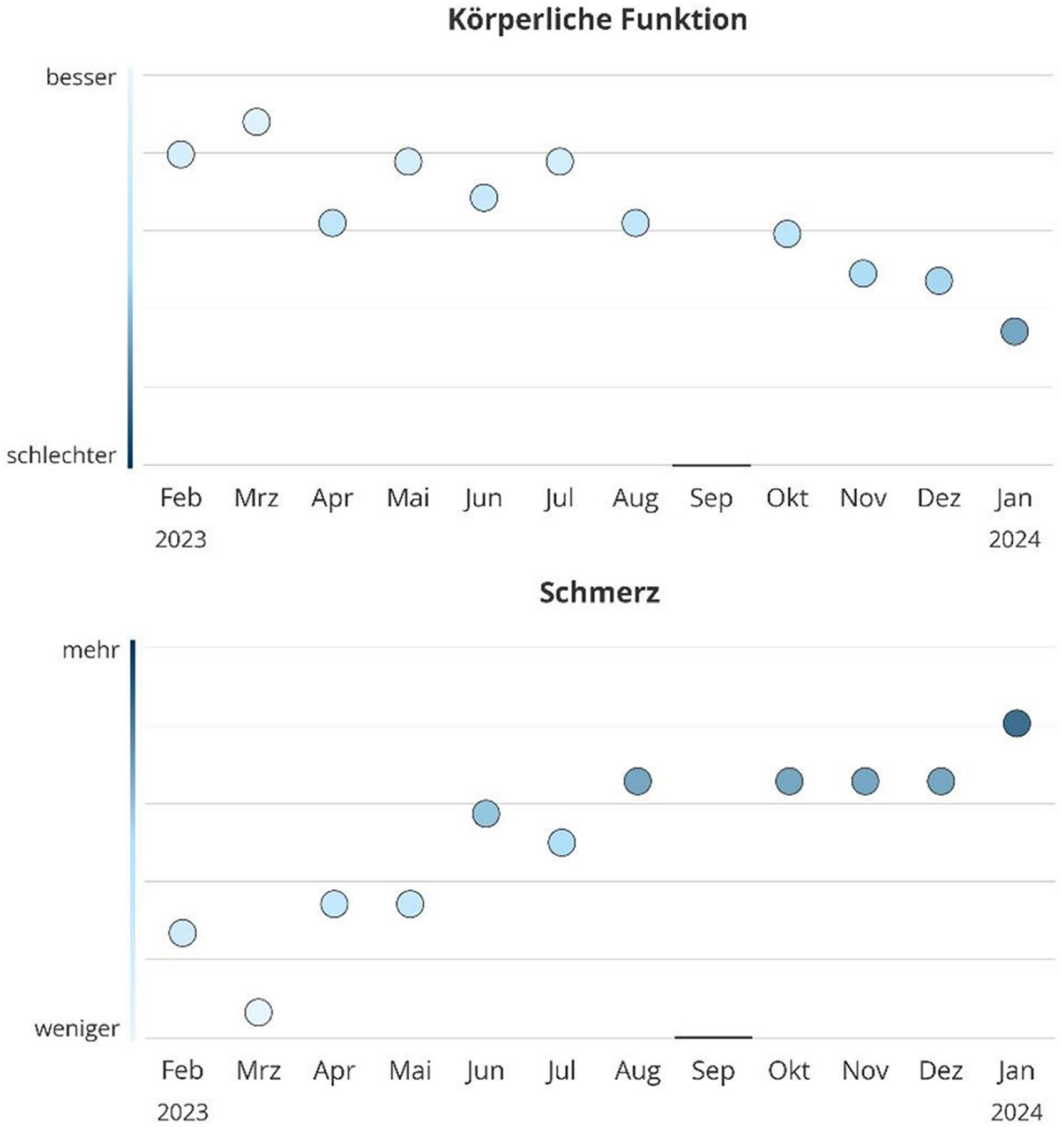
Final design of the PRO visualizations; “Körperliche Funktion” = physical functioning, “besser” = better, “schlechter” = worse; “Schmerz” = pain, “mehr” = more, “weniger” = less, month abbreviations are listed on the x-axis

In collaboration with healthcare service design experts and following a co-design approach with patients and clinical staff from the PRO B study, we developed a visualization concept aimed at being clear, consistent, and emotionally appropriate [12]. The development process was informed by user-centered design principles and incorporated elements from both Experience-Based Co-Design (EBCD) and Design Thinking. Through iterative feedback cycles, visual formats were refined to ensure contextual relevance, usability, and emotional acceptability [14, 15]. Scoring of the EORTC QLQ-C30 followed the official guidelines provided by the EORTC manual, using linear transformation of raw scores to 0–100 scales for all domains [16]. Higher scores represent better functioning on functional scales and greater symptom burden on symptom scales. The final design decisions are summarized in Table 1; an example is given in Fig. 1.

**Table 1:** Design decisions for visualizing PRO data from co-design study with patients and providers

Design Category	Design decision
Chart type	We selected a dot chart, as it allows individual data points to be displayed precisely while keeping the visual layout simple.
	Time is placed on the x-axis to match user expectations.
Color	A single-color blue gradient on the y-axis was chosen over a red–green scale to avoid value judgments, negative emotional associations, and difficulties for users with color vision deficiencies or when printing in greyscale.
	Darker tones indicate more negative values, lighter tones more positive ones, aligning with common mental models; blue was preferred for its neutral and calming associations (see Franconeri et al. [17]).
Labels	All labels are horizontal (8–11 pt), with abbreviated month names and annual markers placed next to the first month of each year.
	For quality-of-life and functioning graphs, the y-axis extremes are labeled “besser” (better) at the top and “schlechter” (worse) at the bottom.
	For symptom graphs, “mehr” (more) is placed at the top and “weniger” (less) at the bottom, reflecting intuitive associations with symptom intensity.
	A vertical color bar on the y-axis reinforces the color gradient meaning, while symbols were avoided to prevent misinterpretation.
Grid lines	For orientation, we included only horizontal grid lines, dividing the graph into five equal areas, while omitting vertical lines to reduce clutter.
Reference data	Reference data are displayed as a subtle background area without a mean line, as the latter could be misleading or cause frustration.
Missing data	Missing data points are marked in a way that ensures visibility without drawing undue attention.
Alert representation	Alerts are shown by marking the data point itself in red, keeping its shape consistent to preserve visual balance.
Item order	The order of all elements follows the EORTC-QLQ-C30 scoring manual.

### Analysis

The cutoff date for receiving evaluation questionnaires was September 26th, 2024. Paper-based answered survey returns were digitized by a student assistant and quality checked by two researchers. Quantitative data were then analyzed descriptively using SPSS Statistics for Mac (Version 30.0.0.0, IBM Corp.) by TP. For the analysis of groups, categorical variables were compared using Pearson’s Chi-squared test. Numeric variables were analyzed using two-sided independent-samples t-tests. In case of non-normally distributed data and survey results based on Likert scales, the Mann-Whitney U test was applied. No formal power calculation was required, as the survey was offered to the entire PRO B study population meeting inclusion criteria at study completion. Figures were produced with MS Office Excel and Miro (Consort Chart).

## Results

**Fig. 2:**
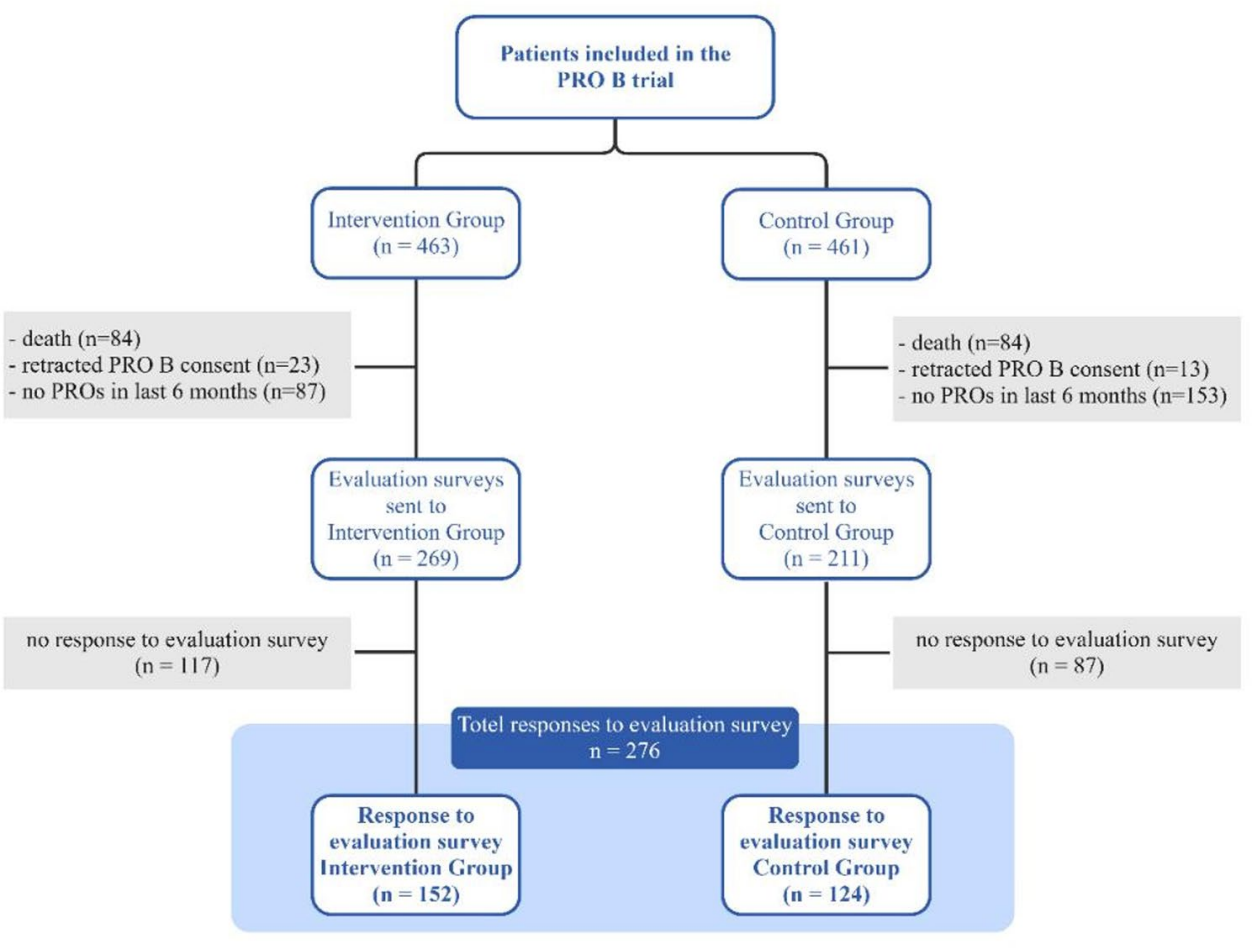
Consort diagram for participants in quantitative evaluation survey

In total, 480 evaluation surveys were sent out to 211 patients in control group and 269 patients in the intervention group. Study participants who died (*n* = 168; intervention group: *n* = 84, control group: *n* = 84) or retracted their consent during the study (*n* = 36; intervention group: *n* = 23, control group: *n* = 13) or who did not answer any questionnaires in the study during the last 6 months (*n* = 240; intervention group: *n* = 87, control group: *n* = 153) were excluded from the evaluation. A total of 276 participants responded to the questionnaire, including 152 from the intervention group and 124 from the control group. A consort diagram is presented in Fig. 2.

Socioeconomic and clinical characteristics of the participating patients at baseline of the PRO B study are reported in Table 2. Groups did differ significantly with regard to the patients’ health status at randomization, with more fully active patients (ECOG 0) but also more severely restricted patients (ECOG > 2) in the intervention group (*p* = 0.005). Also, patients in the intervention group received significantly more chemotherapy at the beginning of PRO B (*p* = 0.03). Socioeconomic parameters were comparable between groups. Missing data of baseline characteristics were minimal and affected less than 1.3% of the dataset (e.g., education level: 1.23%, ECOG performance status: 0.66–0.81%). As these values were missing completely at random and occurred at very low frequency, no imputation was performed. Analyses were conducted based on available cases.

**Table 2:** PRO B baseline characteristics of patients participating in the evaluation survey on PRO visualizations

		Intervention group (*n* = 152)	Control group (*n* = 124)	*P*-value
Mean age in years (range; SD^a^)		52.80 (27–81; SD = 11.12)	52.93 (25–83; SD = 12.11)	0.93
Median time of participation in PRO B in weeks (IQR^b^)		97 (53–112)	91 (65–104)	0.17
Education level n (%)^c^	low	10 (6.58)	11 (8.87)	0.11
	intermediate	74 (48.68)	73 (58.87)	
	high	66 (43.42)	39 (31.45)	
	missing	2 (1.32)	1 (0.81)	
Heath status at randomization (ECOG performance status)^d^ n (%)	0	41 (26.97)	15 (12.10)	0.005
	1	77 (50.66)	85 (68.55)	
	2	27 (17.76)	21 (16.94)	
	> 2	6 (3.95)	2 (1.61)	
	missing	1 (0.66)	1 (0.81)	
HR^e^ status n (%)	HR+/HER- oder HR+/HER+	122 (80.26)	100 (80.65)	0.94
	HR-/HER + oder HR-/HER-	30 (19.74)	24 (19.35)	
Metastases n%)	brain metastases or multiple sites	76 (50.00)	59 (47.58)	0.47
	bone, lymph node, cutaneous metastases	48 (31.58)	47 (37.90)	
	visceral metastases	28 (18.42)	18 (14.52)	
Therapy at randomization n (%)	endocrine therapy	98 (64.47)	85 (68.55)	0.48
	chemotherapy	75 (49.34)	45 (36.29)	0.03
	anti-Her2 therapy	42 (27.63)	38 (30.65)	0.58
	immune therapy	5 (3.29)	3 (2.42)	0.67
	targeted therapy	54 (35.53)	51 (41.13)	0.34
	other therapy	18 (11.84)	13 (10.48)	0.72

^a^ SD = standard devation

^b^ IQR = interquartile range

^c^ Education level according to Destatis classification : low (no/low secondary), intermediate (intermediate secondary), high (upper secondary/university) [18]

^d^ECOG (Eastern Cooperative Oncology Group) performance status: 0 = Fully active, able to carry on all pre-disease performance without restriction, 1 = Restricted in physically strenuous activity but ambulatory and able to carry out work of a light or sedentary nature, e.g., light house work, office work; 2 = Ambulatory and capable of all selfcare but unable to carry out any work activities; up and about more than 50% of waking hours; >2 = only limited selfcare, completely disabled

^e^ HR status = hormone receptor status Only clinically relevant baseline variables are included in Table 2 to reflect factors influencing symptom burden and treatment experience

**Fig. 3:**
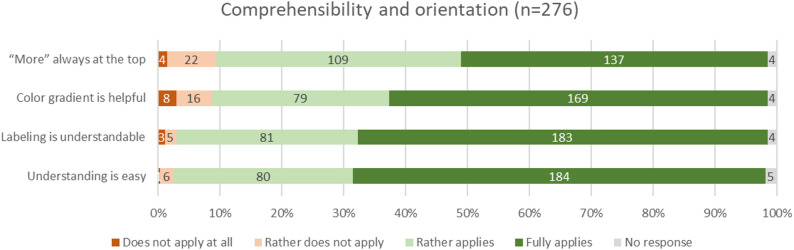
Comprehensibility and orientation of the figures (absolute values and percentages)

**Fig. 4:**
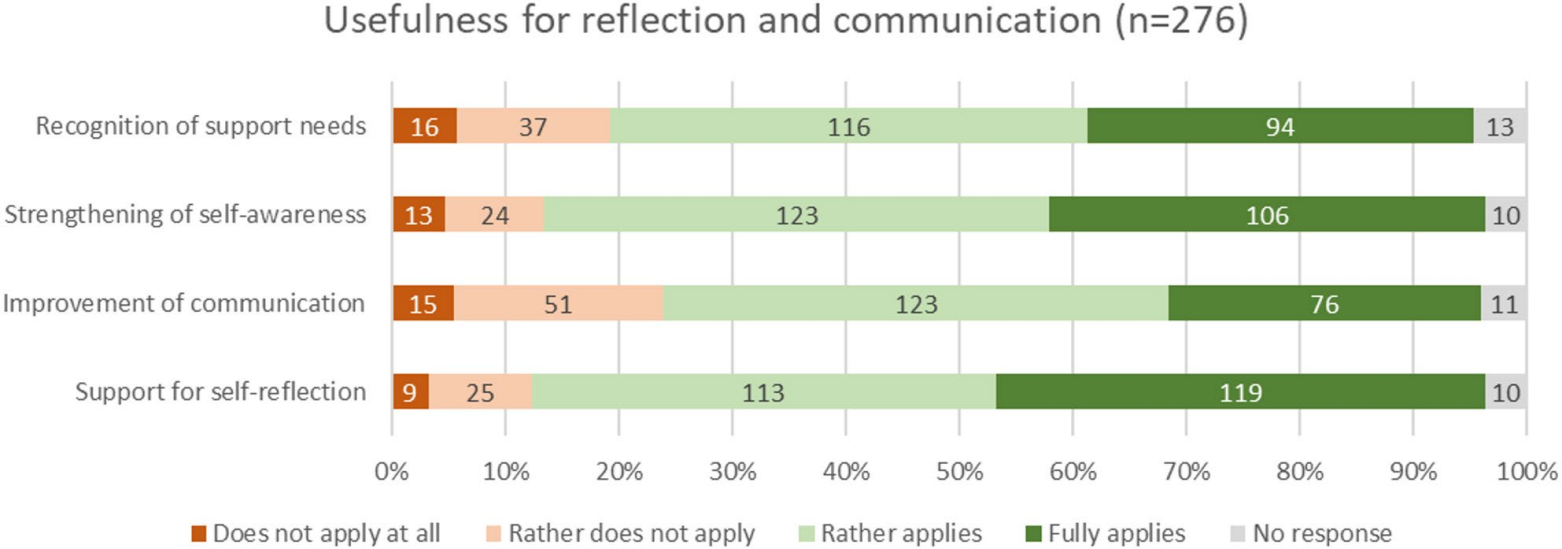
Usefulness of visualizations for reflection and communication (absolute values and percentages)

**Fig. 5:**
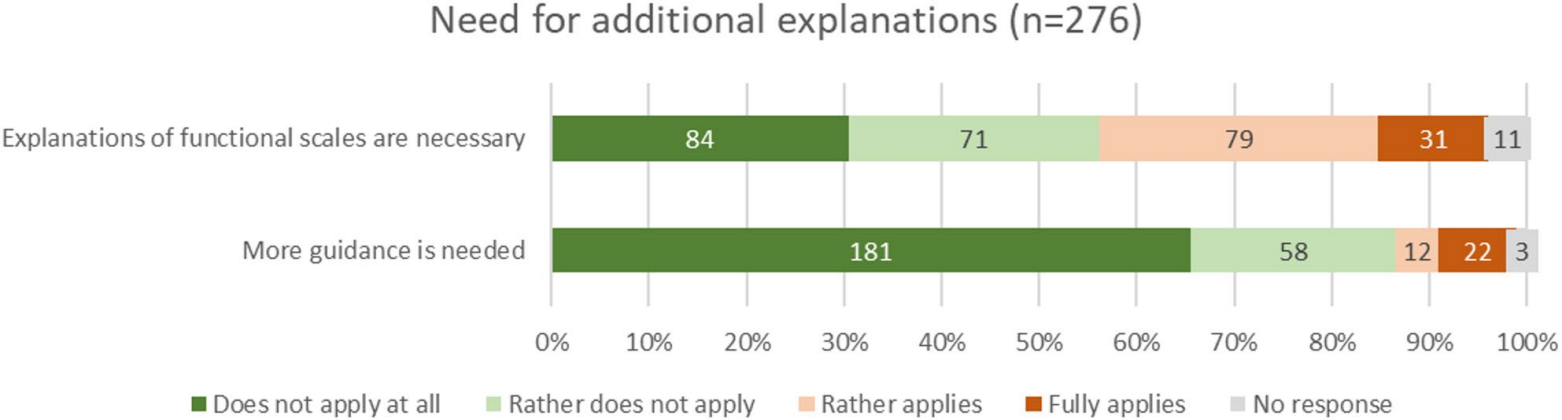
Need for additional explanations in the visualizations (absolute values and percentages)

**Fig. 6:**
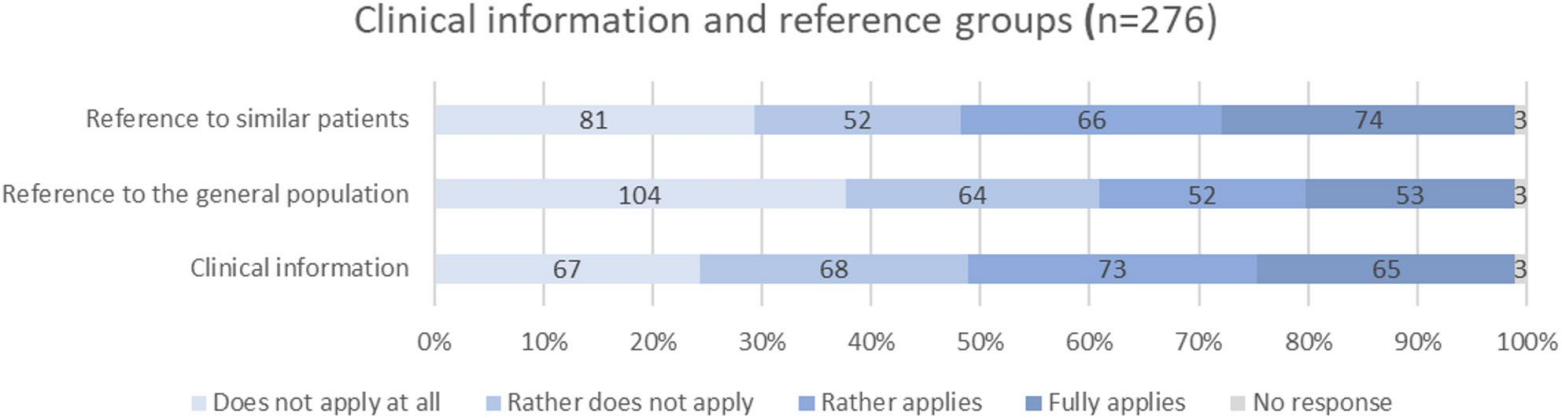
Need for additional clinical information and reference data in the visualizations (absolute values and percentages). Items in this figure are displayed in a neutral color scheme, as they are not directly related to our design decisions but instead reflect potential future functionality of the PRO tool

Survey results were grouped into thematic categories. Positive ratings were defined as responses of “fully applies” or “rather applies,” while negative ratings included “rather does not apply” and “does not apply at all”. Detailed numbers per item are presented in Online Resource 4 (Table 3a) providing the response rates for each answer option by question for intervention and control group. As the intervention and control group differed significantly in only one survey item (the desire for additional clinical information), responses from both groups were aggregated for presentation in Figs. 3, 4, 5 and 6. Missing data of evaluation questions were minimal, affecting on average 2.3% of the dataset (range 0.81–4.83%, median 1.64%). Given the low proportion and absence of systematic patterns, analyses were performed based on available cases.

### Comprehensibility and orientation

Participants rated the visualizations very positively in terms of comprehensibility and orientation of the displayed data. 89.1% of participants (*n* = 246) agreed to the choice of graph directionality positioning high values always at the top end of the x-axis. Similarly, 89.9% of participants (*n* = 248) perceived the color gradient helpful to interpret value expression (dark = negative, light = positive). Even stronger approval was observed for the suggested labeling concept (95.7%, *n* = 264), and the overall comprehensibility of the graphs (96.0%, *n* = 265). Negative ratings were minimal across all items in this category, with less than 10% expressing disagreement. These results indicate a high level of clarity and user-friendliness of the PRO visualizations from the perspective of the participants.

### Usefulness for reflection and communication

Most participants found the PRO visualizations helpful in multiple areas related to understanding their health and interacting with their care team. High levels of agreement were reported for recognizing the need for support (76.1%, *n* = 210), enhancing self-awareness (83.0%, *n* = 229), and encouraging self-reflection (84.1%, *n* = 232). The potential of PRO visualizations to improve communication with healthcare providers (72.1%, *n* = 199) was rated high, but with a bit more skepticism. The PRO reports were thus largely perceived as useful tools for improving the ability to reflect and communicate one’s own health status.

### Need for additional explanations

The results present a mixed picture regarding the need for additional explanations. Explanations for functioning scales, which represent more abstract concepts compared to symptom scales, were rated differently by patients: 56.2% (*n* = 155) saw no need for them, while 39.9% (110) expressed a desire for further information. More extensive guidance within the visualizations was largely rejected (only 12.3% in favor, *n* = 34). Overall, the findings indicate a limited need for additional support, with somewhat greater receptiveness to targeted explanations for more complex content such as functioning scales.

### Additional clinical information and reference groups

The results indicate no clear consensus among participating patients regarding the inclusion of clinical and comparative context such as reference data in PRO visualizations. Opinions on reference data from similar patients were nearly evenly divided, with 50.7% (*n* = 140) in favor and 48.2% (*n* = 133) opposed. In contrast, reference data from the general population were rejected by 60.9% (*n* = 168) of participants, while 38.0% (*n* = 105) expressed support. As for additional clinical information, such as dates of surgery or the start of therapy, 50.0% (*n* = 138) of patients expressed interest, whereas 48.9% (*n* = 135) considered such information unnecessary.

Interestingly, the need for additional clinical context within the PRO visualizations differed significantly between the intervention and control group (*p* < 0.001): Participants in the control group expressed a greater desire for such information (59.7% in favor, *n* = 74 of 124 patients), whereas patients in the intervention group showed no strong preference for these supplementary details (42.1% in favor, *n* = 64 out of 152 patients). No further significant difference occurred between participants that were part of the intervention group and those of the control group in the PRO B trial.

## Discussion

This study quantitatively evaluated newly developed PRO visualizations for metastatic breast cancer care, building on a preceding qualitative co-design process involving patients and providers. Feedback from 276 PRO B participants assessed clarity, usefulness, and potential to enhance patient empowerment through improved self-awareness and communication.

Overall endorsement was high. Participants rated the graphs as comprehensible, orienting, and emotionally approachable. Key design features as consistent directionality (high values at the top), intuitive color gradients (dark = negative), and meaningful axis labels were strongly supported, echoing prior findings that such elements reduce interpretation barriers, especially for patients unfamiliar with raw scores or Tables [7, 19, 20]. Although overall endorsement was high, the risk of social desirability bias is considered low in this context. The survey was conducted anonymously after completion of the main trial, and only a small subset of participants had been involved in the prior co-design phase. Nonetheless, as with any self-reported evaluation, the possibility of biased responses cannot be entirely excluded.

Beyond comprehension, most respondents felt the visualizations supported self-reflection, enhanced health awareness, and facilitated dialogue with their care team, aligning with earlier reports that well-designed PRO tools foster engagement and shared decision-making [21, 22]. Emotional appropriateness was a recurrent strength, avoiding distress from alarming colors or clutter, a consideration emphasized in psycho-oncology literature [23].

Preferences for supplemental information were mixed: fewer than 40% desired additional explanations of abstract PRO domains. Attitudes toward reference data were split, reflecting variability in patient needs depending on health literacy, disease stage, or coping style [7, 24]. These findings suggest that optional, customizable reference displays feasible in digital formats may best accommodate diverse preferences [25].

The only significant difference between PRO B arms concerned additional clinical information (e.g., surgery, treatment): control group patients expressed greater interest than intervention group patients, possibly reflecting the latter’s increased familiarity with their health trajectories from regular PRO engagement, a mechanism consistent with evidence linking repeated self-reporting to improved health literacy ( [1, 26]).

No other group differences emerged, likely due to similar baseline characteristics, limited prior exposure to the new visualizations, and high approval across both arms (possible ceiling effect). These results confirm that co-designed, user-centered PRO visualizations can achieve high acceptance, enhance patient engagement, and remain emotionally appropriate across diverse user groups, supporting their broader integration into oncology care.

While the present quantitative confirmation of our co-designed PRO visualizations provides a strong base for implementation, several issues remain unresolved. The optimal order of PRO domains, whether fixed thematically, prioritized by clinical urgency, or tailored to individual preference, has not been established [20]. The marking of cut-off points and clinically significant changes was not systematically assessed; evidence suggests thresholds can aid detection but may also cause unnecessary alarm if poorly justified [27].

Maintaining consistent directionality across functional (higher = better) and symptom (higher = worse) scales improves interpretability but risks oversimplification [28]. Adaptation to varying health literacy levels remains critical, as lower literacy is associated with reduced benefit from PRO feedback [8]. The selection of an appropriate reference population, general population, disease-specific, or matched by age/treatment, also requires clarification [29].

Finally, this evaluation was limited to paper-based visualizations; interactive digital formats could enable personalization, layered information, and zoom functions, potentially improving engagement and comprehension [30].

To our knowledge, this is one of the first quantitative evaluations of PRO visualizations conducted in a large patient sample. While previous research has predominantly focused on conceptual frameworks, expert guidance, or small-scale qualitative work, the present findings provide empirical insights into patient-perceived clarity, usefulness, and emotional appropriateness of visualized PRO data. This expands on existing evidence by quantifying user preferences and perceived utility in a clearly defined clinical setting - patients with metastatic breast cancer - thereby offering a robust foundation for implementation in similar oncologic contexts.

## Limitations

This study has several limitations. First, although the response rate was high, the evaluation sample may be subject to selection bias, as participation was likely more appealing to patients who were highly motivated and engaged in their care, which needs to be kept in mind regarding the generalizability of these findings. Additionally, the results reflect patient perspectives at a single time point; longitudinal evaluations could offer deeper insights into how perceptions evolve with disease progression or treatment changes. Furthermore, while intervention and control groups were largely comparable, their different levels of exposure to PRO data might have influenced their preferences and familiarity. This aspect should be subject to future research and highlights the importance of tailoring visualizations not only to user characteristics but also to the stage of care and prior experience with PRO monitoring. While baseline characteristics were thoroughly assessed within the responder cohort, no systematic comparison with non-responders was conducted. As a substantial proportion of non-responders had either deceased, withdrawn consent, or were no longer actively engaged at study completion, such analyses would have entailed incomplete data and limited interpretability. This may introduce a different form of potential selection bias, which should be considered when interpreting the generalizability of the findings. Lastly, as this evaluation focused solely on paper-based visualizations, the potential of interactive digital tools, which may offer enhanced personalization and engagement, was not explored.

## Conclusions

The results of this study underscore the value of user-centered visualization in making PRO data more accessible, meaningful, and actionable for patients. The high ratings for clarity and usefulness support the appropriateness of our co-designed visualizations and advocate the integration of these visual tools into routine breast cancer care. However, nuanced aspects such as reference data, context information, and particularly digital interactivity, require continued development and user testing to meet the diverse needs of patients across care settings. The current data provide a fruitful base for developing flexible and interactive digital solutions for establishing PRO data in routine care.

## Supplementary Information

Below is the link to the electronic supplementary material.

Supplementary Material 1

Supplementary Material 2

Supplementary Material 3

Supplementary Material 4

## Data Availability

The experimental and qualitative data that support the findings of this study are included in this published article and its supplementary information files (Online Resources).

## Abbreviations

CG: Control group
DKG: Deutsche Krebsgesellschaft
ECOG: Eastern Cooperative Oncology Group
HR status: Hormone receptor status
IG: Intervention group
IQR: Interquartile range
PRO/PROs: Patient-reported outcomes
SD: Standard devation

## References

[1] Basch E, Deal AM, Kris MG, Scher HI, Hudis CA, Sabbatini P et al (2016) Symptom monitoring with Patient-Reported outcomes during routine cancer treatment: A randomized controlled trial. J Clin Oncol 34(6):557–565 26644527 10.1200/JCO.2015.63.0830 PMC4872028

[2] Denis F, Basch E, Septans AL, Bennouna J, Urban T, Dueck AC et al (2019) Two-Year survival comparing Web-Based symptom monitoring vs routine surveillance following treatment for lung cancer. JAMA 321(3):306–307 30667494 10.1001/jama.2018.18085 PMC6439676

[3] Absolom K, Warrington L, Hudson E, Hewison J, Morris C, Holch P et al (2021) Phase III randomized controlled trial of eRAPID: eHealth intervention during chemotherapy. J Clin Oncol 39(7):734–747 33417506 10.1200/JCO.20.02015

[4] Stillman IO, Boyle B, Lencoski K, Styliadou M, Muir JM, Sarri G (2025) Rooting patient-reported outcomes in clinical care: a scoping review on benefits, challenges, and opportunities for patients and clinicians. Health Qual Life Outcomes 23(1):93 41024025 10.1186/s12955-025-02430-7 PMC12482245

[5] Bantug ET, Coles T, Smith KC, Snyder CF, Rouette J, Brundage MD et al (2016) Graphical displays of patient-reported outcomes (PRO) for use in clinical practice: what makes a pro picture worth a thousand words? Patient Educ Couns 99(4):483–490 26603445 10.1016/j.pec.2015.10.027

[6] McNair AG, Brookes ST, Davis CR, Argyropoulos M, Blazeby JM (2010) Communicating the results of randomized clinical trials: do patients understand multidimensional patient-reported outcomes? J Clin Oncol 28(5):738–743 20065187 10.1200/JCO.2009.23.9111

[7] Albers EAC, Fraterman I, Walraven I, Wilthagen E, Schagen SB, van der Ploeg IM et al (2022) Visualization formats of patient-reported outcome measures in clinical practice: a systematic review about preferences and interpretation accuracy. J Patient Rep Outcomes 6(1):18 35239055 10.1186/s41687-022-00424-3 PMC8894516

[8] McCaffery KJ, Holmes-Rovner M, Smith SK, Rovner D, Nutbeam D, Clayman ML et al (2013) Addressing health literacy in patient decision aids. BMC Med Inf Decis Mak 13(Suppl 2):S10 10.1186/1472-6947-13-S2-S10 PMC4042520 24624970

[9] Sorensen K, Van den Broucke S, Fullam J, Doyle G, Pelikan J, Slonska Z et al (2012) Health literacy and public health: a systematic review and integration of definitions and models. BMC Public Health 12:80 22276600 10.1186/1471-2458-12-80 PMC3292515

[10] Elsman EBM, Boers M, Terwee CB, Beaton D, Abma I, Aiyegbusi OL et al (2025) Systematic reviews of patient-reported outcome measures (PROMs): table templates for effective communication. Qual Life Res 10.1007/s11136-025-04058-y PMC12689657 40900239

[11] Karsten MM, Kuhn F, Pross T, Blohmer JU, Hage AM, Fischer F et al (2021) PRO B: evaluating the effect of an alarm-based patient-reported outcome monitoring compared with usual care in metastatic breast cancer patients-study protocol for a randomised controlled trial. Trials 22(1):666 34583744 10.1186/s13063-021-05642-6 PMC8479993

[12] Doppelbauer L, Karsten MM, Tatzber A, Hatzler L, Gebert P, Brands J et al (2025) From participatory design to clinical routine: new concepts for visualizing patient-reported outcomes in breast cancer care. Manuscript submitted for publication 10.1186/s41687-025-00984-0 PMC12830510 41422188

[13] Gebert P, Karsten MM, Hage AM, Dordevic AD, Grittner U (2024) Statistical analysis plan for the PRO B study: open-label, superiority randomised controlled trial of alarm-based patient-reported outcome monitoring in patients with metastatic breast cancer. Trials. 25(1) 10.1186/s13063-024-08025-9 PMC10918931 38448904

[14] Bate P, Robert G (2006) Experience-based design: from redesigning the system around the patient to co-designing services with the patient. Qual Saf Health Care 15(5):307–310 17074863 10.1136/qshc.2005.016527 PMC2565809

[15] Brown T, Katz B (2009) Change by design: how design thinking transforms organizations and inspires innovation. Harper Business

[16] Fayers P, Bottomley A (2002) Quality of life research within the EORTC-the EORTC QLQ-C30. European organisation for research and treatment of cancer. Eur J Cancer 38(Suppl 4):S125–S133 11858978 10.1016/s0959-8049(01)00448-8

[17] Franconeri SL, Padilla LM, Shah P, Zacks JM, Hullman J (2021) The science of visual data communication: what works. Psychol Sci Public Interest 22(3):110–161 34907835 10.1177/15291006211051956

[18] Destatis SB (2025) Bildungsstand: Statistisches Bundesamt, Gustav-Stresemann-Ring 11, 65189 Wiesbaden; Available from: https://www.destatis.de/DE/Themen/Gesellschaft-Umwelt/Einkommen-Konsum-Lebensbedingungen/Glossar/bildung-zve.html

[19] Fischer KI, De Faoite D, Rose M (2020) Patient-reported outcomes feedback report for knee arthroplasty patients should present selective information in a simple design - findings of a qualitative study. J Patient Rep Outcomes 4(1):6 31965364 10.1186/s41687-020-0173-7 PMC6973599

[20] Brundage MD, Smith KC, Little EA, Bantug ET, Snyder CF, Board PRODPSA (2015) Communicating patient-reported outcome scores using graphic formats: results from a mixed-methods evaluation. Qual Life Res 24(10):2457–2472 26012839 10.1007/s11136-015-0974-y PMC4891942

[21] Greenhalgh J, Gooding K, Gibbons E, Dalkin S, Wright J, Valderas J et al (2018) How do patient reported outcome measures (PROMs) support clinician-patient communication and patient care? A realist synthesis. J Patient Rep Outcomes 2:42 30294712 10.1186/s41687-018-0061-6 PMC6153194

[22] Snyder CF, Jensen RE, Segal JB, Wu AW (2013) Patient-reported outcomes (PROs): putting the patient perspective in patient-centered outcomes research. Med Care 51(8 Suppl 3):S73–S79 23774513 10.1097/MLR.0b013e31829b1d84 PMC3771694

[23] McCorkle R, Ercolano E, Lazenby M, Schulman-Green D, Schilling LS, Lorig K et al (2011) Self-management: enabling and empowering patients living with cancer as a chronic illness. CA Cancer J Clin 61(1):50–62 21205833 10.3322/caac.20093 PMC3058905

[24] Katz P, Dall’Era M, Trupin L, Rush S, Murphy LB, Lanata C et al (2021) Impact of limited health literacy on Patient-Reported outcomes in systemic lupus erythematosus. Arthritis Care Res (Hoboken) 73(1):110–119 32741118 10.1002/acr.24361 PMC7775267

[25] Shahid R, Shoker M, Chu LM, Frehlick R, Ward H, Pahwa P (2022) Impact of low health literacy on patients’ health outcomes: a multicenter cohort study. BMC Health Serv Res 22(1):1148 36096793 10.1186/s12913-022-08527-9 PMC9465902

[26] Kotronoulas G, Kearney N, Maguire R, Harrow A, Di Domenico D, Croy S et al (2014) What is the value of the routine use of patient-reported outcome measures toward improvement of patient outcomes, processes of care, and health service outcomes in cancer care? A systematic review of controlled trials. J Clin Oncol 32(14):1480–1501 24711559 10.1200/JCO.2013.53.5948

[27] Breidenbach C, Kowalski C, Wesselmann S, Sibert NT (2021) Could existing infrastructure for using patient-reported outcomes as quality measures also be used for individual care in patients with colorectal cancer? BMC Health Serv Res 21(1):448 33975586 10.1186/s12913-021-06457-6 PMC8111716

[28] Snyder C, Smith K, Holzner B, Rivera YM, Bantug E, Brundage M et al (2019) Making a picture worth a thousand numbers: recommendations for graphically displaying patient-reported outcomes data. Qual Life Res 28(2):345–356 30306533 10.1007/s11136-018-2020-3 PMC6363861

[29] Thanarajasingam G, Bhatnagar V, Noble BN, Chen TY, Fiero MH, Hoffman R et al (2025) Longitudinal graphics of patient-reported physical function in patients treated for hematologic malignancies. BMC Med Res Methodol 25(1):189 40775758 10.1186/s12874-025-02617-y PMC12329971

[30] Wu AW, Kharrazi H, Boulware LE, Snyder CF (2013) Measure once, cut twice–adding patient-reported outcome measures to the electronic health record for comparative effectiveness research. J Clin Epidemiol 66(8 Suppl):S12–20 23849145 10.1016/j.jclinepi.2013.04.005 PMC3779680

