# Supplementary material for: Quantitative assessment of visual designs for communicating patient-reported outcomes in breast cancer care to patients
Source: J Patient Rep Outcomes. 2025 Dec 20;10:12. doi: 10.1186/s41687-025-00984-0 (PMC12830510; doi:10.1186/s41687-025-00984-0)
Supplement: Supplementary file 1 — Supplementary Material 1 [file 41687_2025_984_MOESM1_ESM.pdf]

Liebe Patientin,

zum Abschluss der PRO B Studie möchten wir uns herzlich für Ihre Teilnahme bedanken und ein letztes Mal um Ihre Mithilfe bitten:

Die PRO B Studie untersuchte, wie sich eine digitale Betreuung mit Befragungen auf das Befinden und den Krankheitsverlauf von Patientinnen auswirkt. Nach der Auswertung wird entschieden, ob diese digitale Betreuung in Zukunft deutschlandweit für Brustkrebspatientinnen angeboten werden sollte.

Sie wurden wöchentlich per App befragt, und im Falle einer Verschlechterung von Befragungswerten erhielten Sie einen Anruf von Ihrem Behandlungsteam.

Eine Übersicht Ihrer Befragungsergebnisse der PRO B Studie stellen wir Ihnen beiliegend zur Verfügung.

Als Studienteilnehmerin ist Ihre persönliche Einschätzung von unschätzbarem Wert. Durch Ihr Feedback helfen Sie uns, die digitale Betreuung wie in der PRO B Studie weiterzuentwickeln. Wir bitten Sie daher, anhand der nachfolgenden Fragen und Aussagen die PRO B Studie (Teil A) und die Darstellung der Befragungsergebnisse zu bewerten (Teil B):

Teil A: Bewertung der PRO B Studie

1. **Allgemeine Bewertung der Fragebögen:**

- Wie **lange** haben Sie in der Regel etwa gebraucht, um einen Wochen-Fragebogen in der App auszufüllen? → Etwa ________ Minuten

Bitte bewerten Sie die folgenden Aussagen auf einer Skala von „trifft überhaupt nicht zu“ bis „trifft vollkommen zu“. Setzen Sie hierzu pro Aussage **ein** Kreuz.

	Trifft überhaupt nicht zu	Trifft eher nicht zu	Trifft eher zu	Trifft vollkommen zu
Die Länge der Fragebögen war für mich angemessen.	☐	☐	☐	☐
Die Fragen und Antwortmöglichkeiten waren verständlich für mich.	☐	☐	☐	☐
Durch das Ausfüllen der Fragebögen habe ich besser erkannt, wie es mir wirklich geht.	☐	☐	☐	☐
Die Fragen in den Fragebögen waren insgesamt relevant für mich.	☐	☐	☐	☐

- Fehlten aus Ihrer Sicht inhaltliche Aspekte im Fragebogen, die für Sie ebenfalls relevant gewesen wären?

  ☐ Nein

  ☐ Ja, folgende: ____

2. Bewertung der Anrufe in der PRO B Studie:

Wurde eine relevante Verschlechterung in Ihren Antworten in den Fragebögen gemessen, dann hat Ihr Behandlungsteam Sie telefonisch kontaktiert. Zu diesen Anrufen der PRO B Studie möchten wir Sie näher befragen.

- Haben Sie **mindestens einmal einen Anruf** im Rahmen der PRO B Studie erhalten?

  ☐ Nein → *bitte überspringen Sie dieses Fragenkapitel und gehen zu Kapitel 3. auf S. 3*

  ☐ Ja

- Bei den Anrufen der PRO B Studie hatte ich in der Regel eine feste Ansprechpartnerin/ einen festen Ansprechpartner.

  ☐ meistens

  ☐ manchmal

  ☐ nie

  ☐ ich erinnere mich nicht mehr

Bitte bewerten Sie die folgenden Aussagen auf einer Skala von „trifft überhaupt nicht zu“ bis „trifft vollkommen zu“. Setzen Sie hierzu pro Aussage **ein** Kreuz.

	Trifft über-haupt nicht zu	Trifft eher nicht zu	Trifft eher zu	Trifft voll-kommen zu
Bei den Anrufen zur PRO B Studie hatte ich ausreichend Gelegenheit, um über mein Befinden zu sprechen.	☐	☐	☐	☐
Bei den Anrufen zur PRO B Studie fühlte ich mich ernst genommen.	☐	☐	☐	☐
Ich habe mich durch die Anrufe zur PRO B Studie zusätzlich unterstützt gefühlt.	☐	☐	☐	☐
Wenn ich Anrufe zur PRO B Studie erhielt, lag auch tatsächlich eine Verschlechterung bei mir vor.	☐	☐	☐	☐
Ich hatte keinen Bedarf für den zusätzlichen Kontakt durch die PRO B Anrufe.	☐	☐	☐	☐
Die Anrufe zur PRO B Studie waren zu häufig.	☐	☐	☐	☐
Mich haben die Anrufe zur PRO B Studie gestört.	☐	☐	☐	☐

Ferner möchten wir herausfinden, ***wie*** Sie in Ihren Anrufen zur PRO B Studie unterstützt wurden. Bitte setzen Sie hierzu pro Aussage **ein** Kreuz.

**In den Anrufen zur PRO B Studie …**	Trifft nicht zu	Trifft zu
… wurden mir zusätzliche medizinische Informationen zu meiner Erkrankung und Therapie vermittelt.	☐	☐
… wurden mir Empfehlungen zum Umgang mit Nebenwirkungen gegeben.	☐	☐
… wurden mir soziale/administrative Informationen vermittelt, z.B. zur Pflegegradbeantragung oder Verschreibung von Hilfsmitteln.	☐	☐
… wurden mir Kontakte zu anderen unterstützenden Anlaufstellen weitergegeben.	☐	☐
… wurde ich bei Terminvereinbarungen unterstützt.	☐	☐
… wurde mir mindestens einmal empfohlen in die Rettungsstelle zu kommen.	☐	☐
Mir wurde anderweitig durch die Anrufe der PRO B Studie geholfen (Freitext):		

3. Bewertung Ihrer Teilnahme an der PRO B Studie:

Des Weiteren möchten wir herausfinden, wie Ihre Teilnahme an der PRO B Studie Ihre Betreuung beeinflusst hat. Bitte setzen Sie hierzu pro Aussage **ein** Kreuz.

**Durch meine Teilnahme an der PRO B Studie…**	Trifft überhaupt nicht zu	Trifft eher nicht zu	Trifft eher zu	Trifft vollkommen zu
… war ich häufiger in Kontakt mit meinem Behandlungsteam als vor der Studie.	☐	☐	☐	☐
… war ich schneller in Kontakt mit meinem Behandlungsteam als vor der Studie.	☐	☐	☐	☐
… konnte ich dem Behandlungsteam meinen Gesundheitszustand besser mitteilen als vor der Studie.	☐	☐	☐	☐
… hatte ich ein zusätzliches Sicherheitsgefühl.	☐	☐	☐	☐
Die Teilnahme an der PRO B Studie hatte insgesamt einen Mehrwert für mich.	☐	☐	☐	☐

4. Langfristige Perspektiven auf die PRO B Studie:

- Würden Sie sich wünschen, dass die digitale Betreuung aus der PRO B Studie deutschlandweit als Teil der regulären Versorgung für alle Patientinnen mit fortgeschrittenem Brustkrebs angeboten werden würde?

  ☐ Ja

  ☐ Nein

  - Ggf. Gründe:______________________________

- Können Sie sich vorstellen, weiter langfristig an einer Befragung mit Anrufen bei Verschlechterung (wie in der PRO B Studie) teilzunehmen?

  ☐ Ja, wöchentlich

  ☐ Ja, aber Befragung in anderen Abständen:

  - täglich
  - mehrmals in der Woche
  - alle 2 Wochen
  - monatlich
  - alle 3 Monate
  - nur bei Bedarf

  ☐ Nein, überhaupt nicht

  - Ggf. Gründe:______________________________

- Haben Sie Verbesserungsvorschläge oder Anregungen für das Konzept der PRO B Studie? (Freitext)

Teil B: Bewertung der Abbildungen Ihrer Befragungsergebnisse

Die folgenden Aussagen beziehen sich auf die Abbildungen zu Ihren Symptomen und zu Ihrer Funktionsfähigkeit, die Ihre Befragungsergebnisse in der PRO B Studie widerspiegeln (siehe S. R 1 – R 7). Bitte bewerten Sie die folgenden Aussagen auf einer Skala von „trifft überhaupt nicht zu“ bis „trifft vollkommen zu“. Setzen Sie pro Aussage **ein** Kreuz.

5. **Bewertung der Verständlichkeit der Abbildungen:**

	Trifft über-haupt nicht zu	Trifft eher nicht zu	Trifft eher zu	Trifft voll-kommen zu
Es fällt mir leicht, meine Befragungsergebnisse zu verstehen.	☐	☐	☐	☐
Ich benötige mehr Hilfestellungen, um meine Befragungsergebnisse zu verstehen.	☐	☐	☐	☐
Die Beschriftung der Abbildungen ist klar und verständlich.	☐	☐	☐	☐
Dass manche Punkte heller und manche Punkte dunkler dargestellt sind, hilft mir zu verstehen, wo die Befragungsergebnisse besser und wo schlechter sind.	☐	☐	☐	☐
Ich benötige die Erläuterungen zu den Abbildungen meiner Funktionsfähigkeit (grauer Kasten), um zu verstehen, was unter den Funktionsfähigkeiten (z.B. Emotionale Funktion, Körperliche Funktion) zu verstehen ist.	☐	☐	☐	☐
Eine Darstellung von Befragungsergebnissen, bei der „mehr“ immer oben ist, egal ob mehr Symptome oder mehr Funktionsfähigkeit, finde ich verständlich.	☐	☐	☐	☐
Ich möchte innerhalb der Abbildungen gern Verweise auf meine wichtigen klinischen Informationen wie z.B. mein Operationsdatum oder Therapiebeginn sehen.	☐	☐	☐	☐
Ich möchte innerhalb der Abbildungen gern zusätzlich zu meinen Befragungsergebnissen die Vergleichswerte der gesunden Bevölkerung sehen.	☐	☐	☐	☐
Ich möchte innerhalb der Abbildungen zusätzlich zu meinen Befragungsergebnissen die Vergleichswerte von Patientinnen sehen, die mir in Erkrankung und allgemeinem Gesundheitsstatus möglichst ähnlich sind.	☐	☐	☐	☐

6. Möglicher Nutzen dieser Abbildungen für Ihre Betreuung:

	Trifft überhaupt nicht zu	Trifft eher nicht zu	Trifft eher zu	Trifft vollkommen zu
Die Befragungsergebnisse helfen mir, den Verlauf meines Gesundheits- bzw. Krankheitszustands zu reflektieren.	☐	☐	☐	☐
Die Abbildungen haben das Potenzial, meine Kommunikation mit dem Gesundheitspersonal über den Verlauf meines Gesundheits- bzw. Krankheitszustands zu erleichtern.	☐	☐	☐	☐
Durch die Abbildung der Befragungsergebnisse fühle ich mich bestärkt darin, wie ich meinen Gesundheits- und Krankheitszustand wahrnehme.	☐	☐	☐	☐
Durch die Abbildung der Befragungsergebnisse verstehe ich besser, in welchen Bereichen ich Unterstützung benötige.	☐	☐	☐	☐

Vielen Dank für Ihre wertvolle Perspektive und Ihre Teilnahme an der Bewertung der PRO B Studie. Damit tragen Sie dazu bei, die zukünftige Betreuung und Versorgung von Brustkrebspatientinnen mitzugestalten.
