# Supplementary material for: Quantitative assessment of visual designs for communicating patient-reported outcomes in breast cancer care to patients
Source: J Patient Rep Outcomes. 2025 Dec 20;10:12. doi: 10.1186/s41687-025-00984-0 (PMC12830510; doi:10.1186/s41687-025-00984-0)
Supplement: Supplementary file 2 — Supplementary Material 2 [file 41687_2025_984_MOESM2_ESM.pdf]

Studienzentrum

Patient-ID

Erläuterung zur Interpretation der Befragungsergebnisse

Auf den nächsten Seiten sehen Sie für die letzten 12 Monate der PRO B Studie den Verlauf Ihrer Ergebnisse zu Lebensqualität & Funktionsfähigkeit und zu Ihren Symptomen. Auf der unteren Achse ist die Zeit in Monaten abgebildet. Zur besseren Übersicht haben wir für jeden Monat einen Mittelwert Ihrer wöchentlichen Antworten zusammengefasst.

Auf der linken Achse sehen Sie einen Farbverlauf. Für Symptome entspricht ein helles Blau weniger Symptomen und ein dunkles Blau mehr Symptomen. Für Lebensqualität & Funktionsfähigkeit entspricht ein helles Blau besserer Funktionsfähigkeit und ein dunkles Blau schlechterer Funktionsfähigkeit.

Wenn Sie Fragebögen für mehr als 12 Monate beantwortet haben und auch länger zurückliegende Ergebnisse erhalten möchten, wenden Sie sich bitte an pro-b-projekt@charite.de.

Lebensqualität beschreibt Ihr subjektives Wohlbefinden im Alltag.

Körperliche Funktion beschreibt, wie gut Ihr Körper den Alltag bewältigen kann (z.B. einen längeren Spaziergang machen).

Rollenfunktion bezieht sich darauf, wie gut Sie sich in der Lage sehen, alltägliche Aufgaben zu erfüllen und an Freizeitaktivitäten teilzunehmen.

Emotionale Funktion bezieht sich darauf, wie stark Sie Sorgen oder Gefühle wie Niedergeschlagenheit oder Reizbarkeit wahrnehmen.

Kognitive Funktion bezieht sich auf Ihre Konzentrationsfähigkeit und Aufmerksamkeitsleistung sowie Ihr Erinnerungsvermögen.

Soziale Funktion beschreibt, wie Sie Ihre Beziehungen zu anderen Menschen wahrnehmen und wie aktiv Sie an gemeinsamen Unternehmungen in Ihrem sozialen Umfeld teilnehmen.

Diese Seite wurde bewusst leer gelassen.

Schmerz

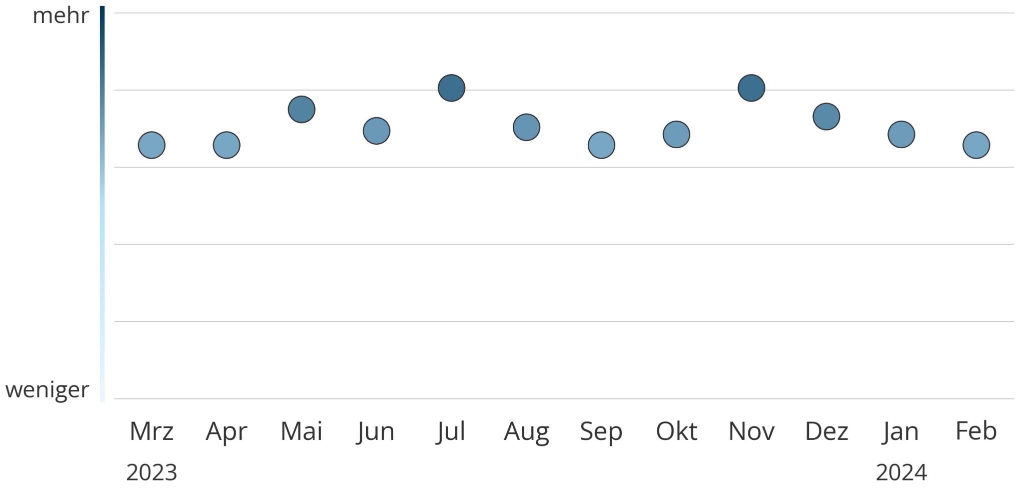

Atemnot

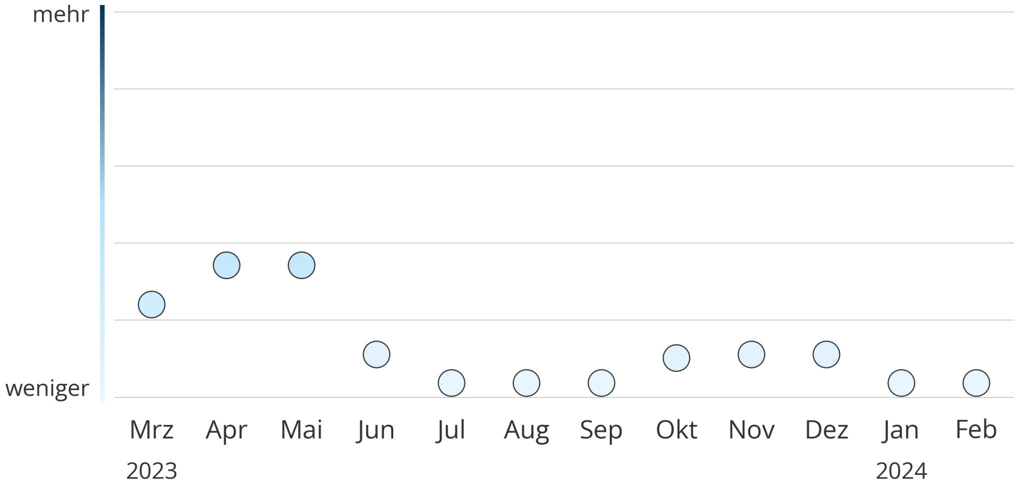

Fatigue (Erschöpfung)

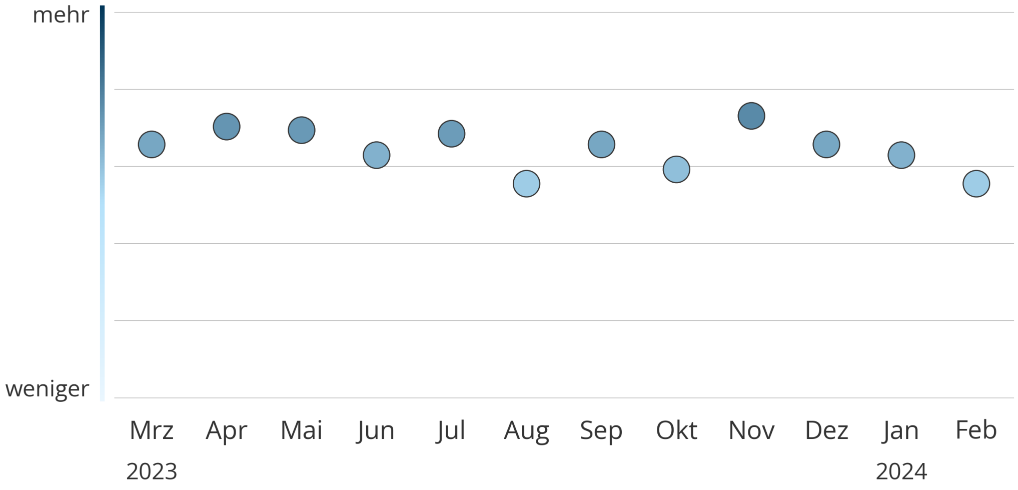

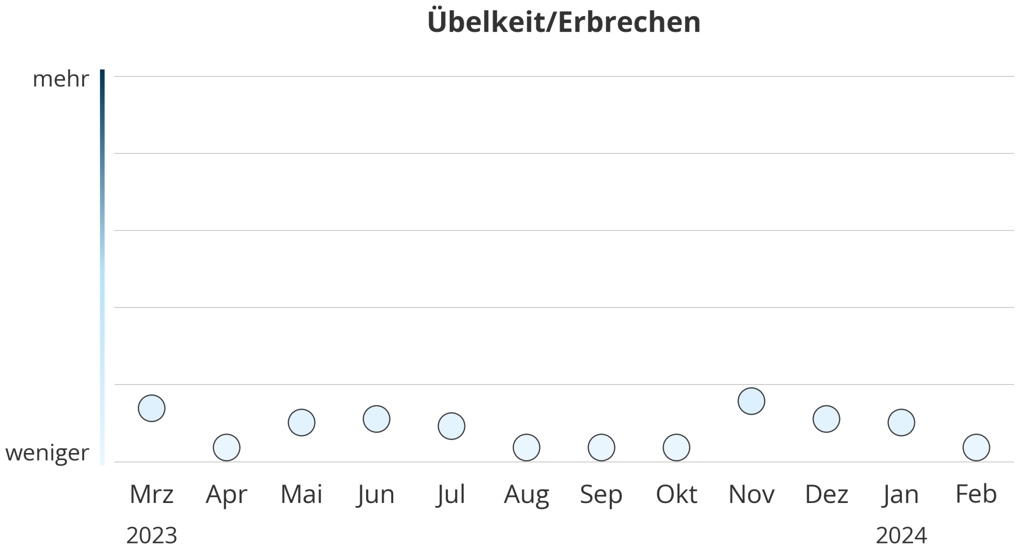
Übelkeit/Erbrechen
mehr
weniger
Mrz
2023
Apr
Mai
Jun
Jul
Aug
Sep
Okt
Nov
Dez
Jan
2024
Feb

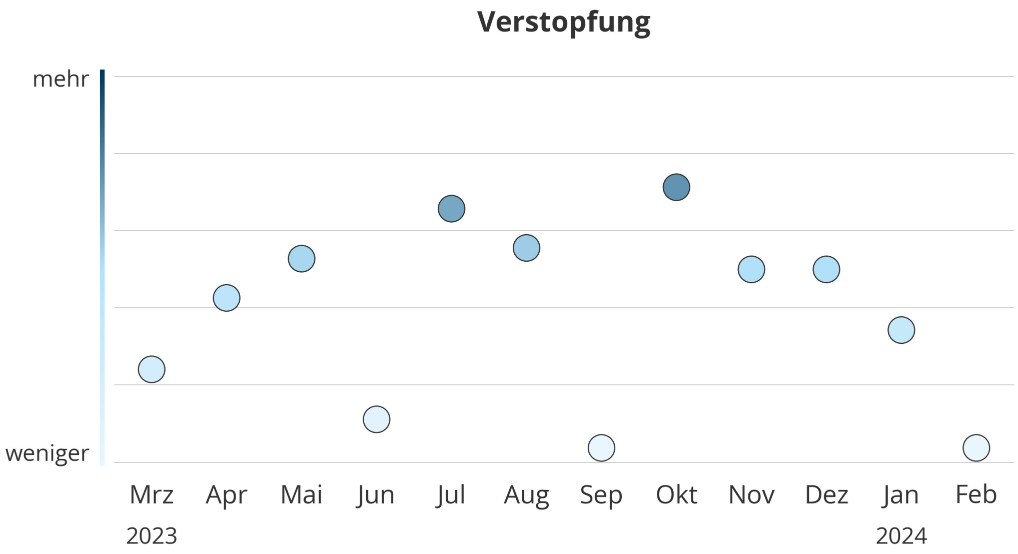
Verstopfung
mehr
weniger
Mrz
2023
Apr
Mai
Jun
Jul
Aug
Sep
Okt
Nov
Dez
Jan
2024
Feb

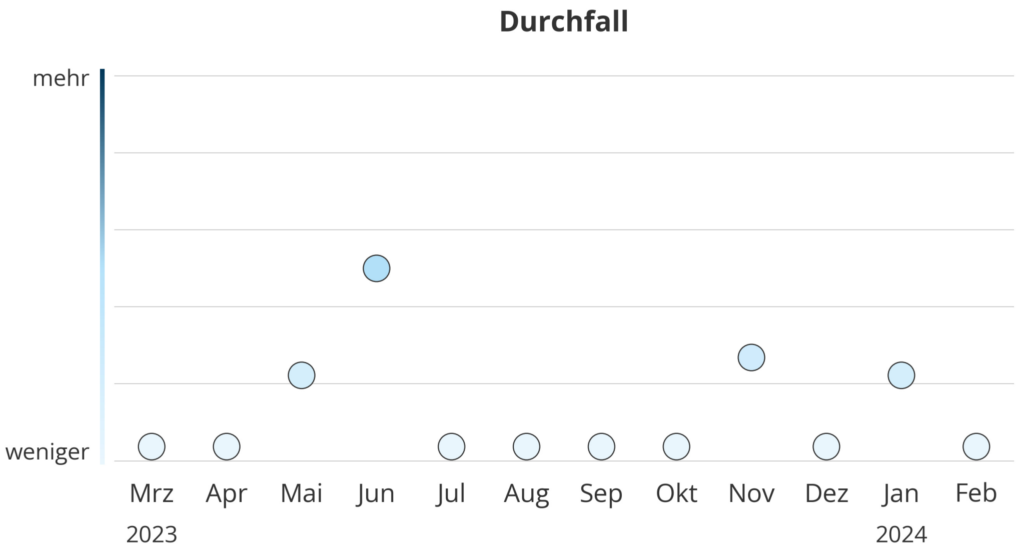
Durchfall
mehr
weniger
Mrz
2023
Apr
Mai
Jun
Jul
Aug
Sep
Okt
Nov
Dez
Jan
2024
Feb

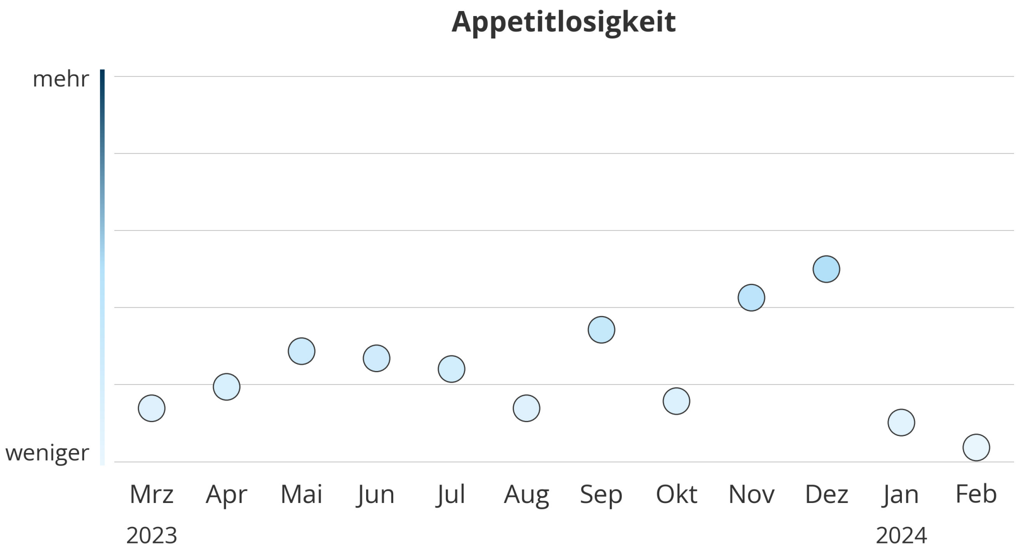
Appetitlosigkeit
mehr
weniger
Mrz
Apr
Mai
Jun
Jul
Aug
Sep
Okt
Nov
Dez
Jan
Feb
2023
2024

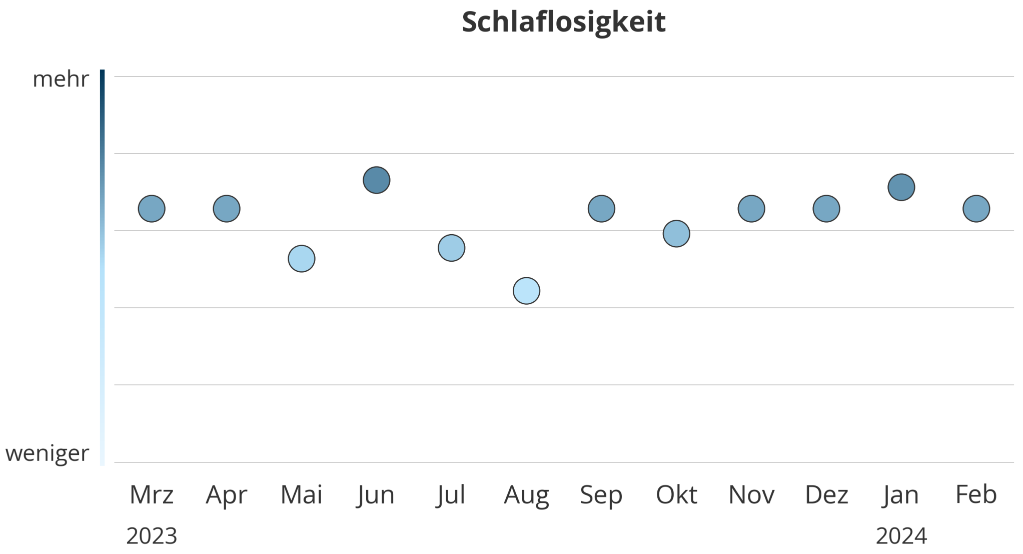
Schlaflosigkeit
mehr
weniger
Mrz
Apr
Mai
Jun
Jul
Aug
Sep
Okt
Nov
Dez
Jan
Feb
2023
2024

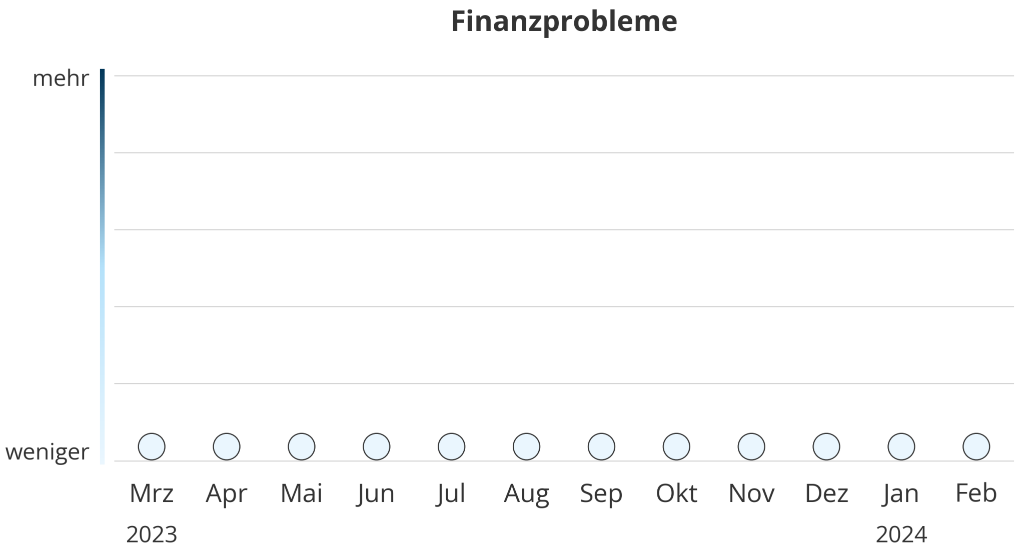
Finanzprobleme
mehr
weniger
Mrz
Apr
Mai
Jun
Jul
Aug
Sep
Okt
Nov
Dez
Jan
Feb
2023
2024

Lebensqualität

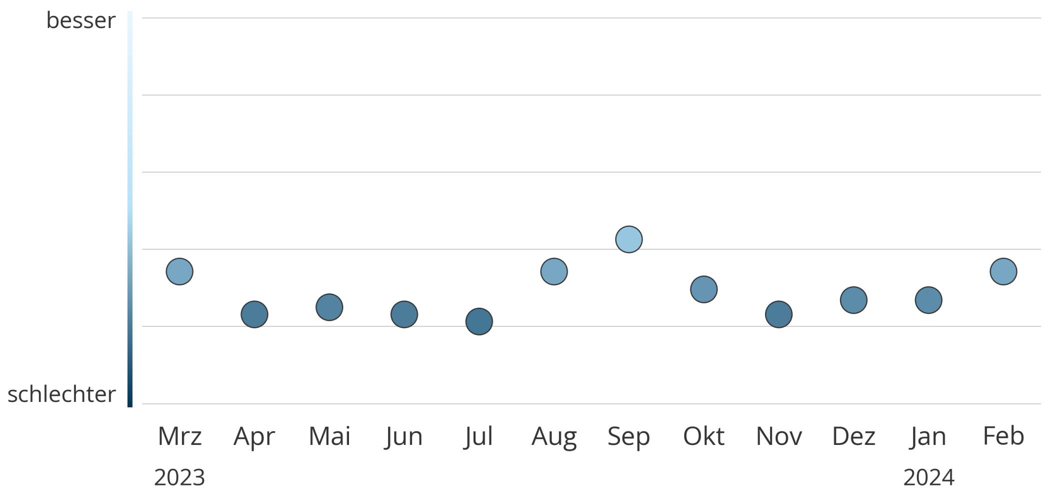

Körperliche Funktion

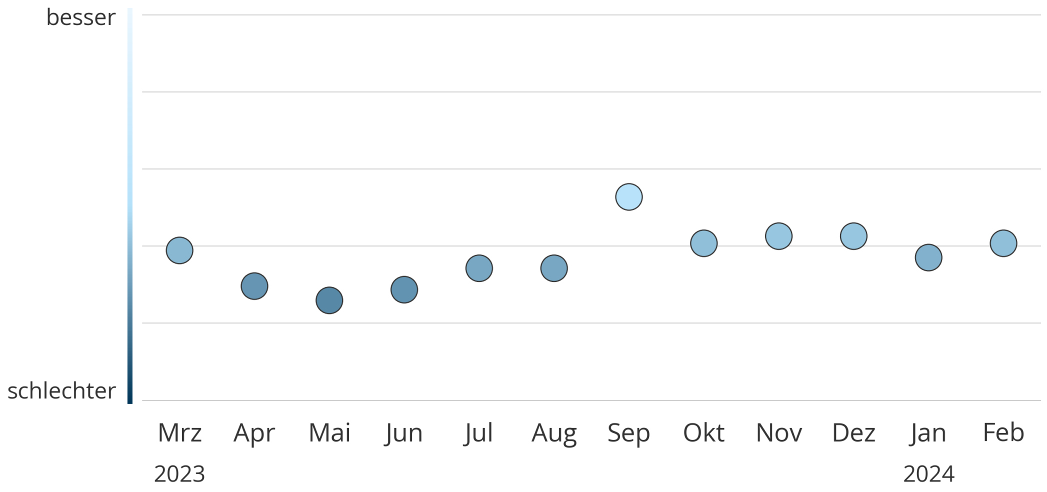

Rollenfunktion

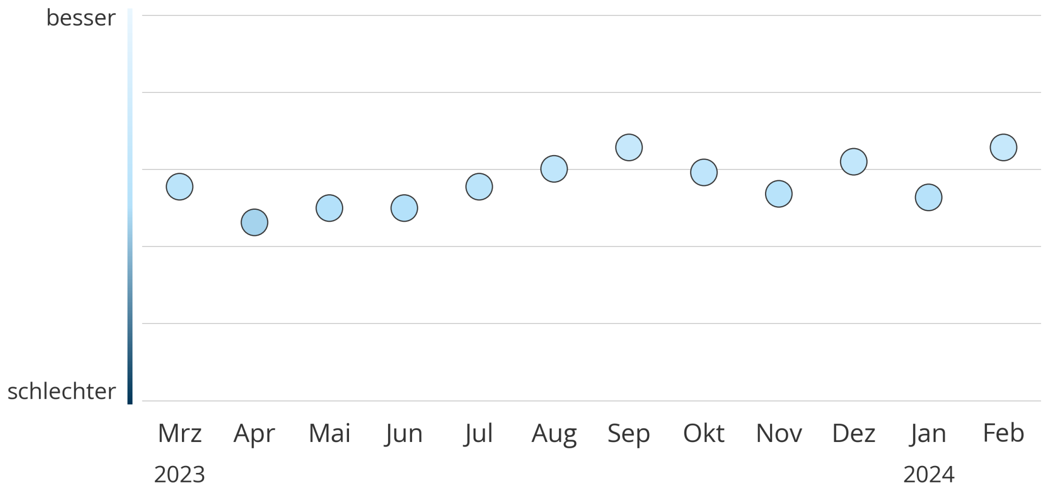

Emotionale Funktion

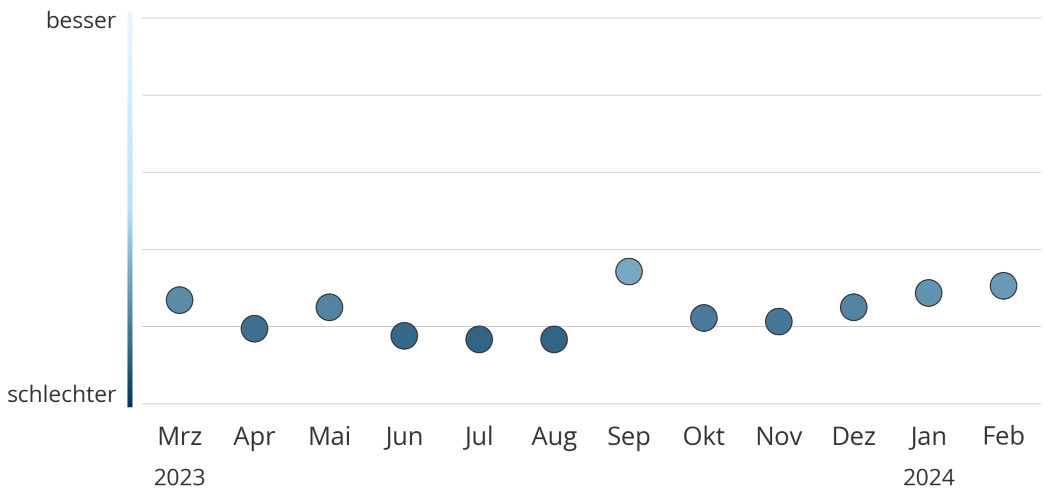

Kognitive Funktion

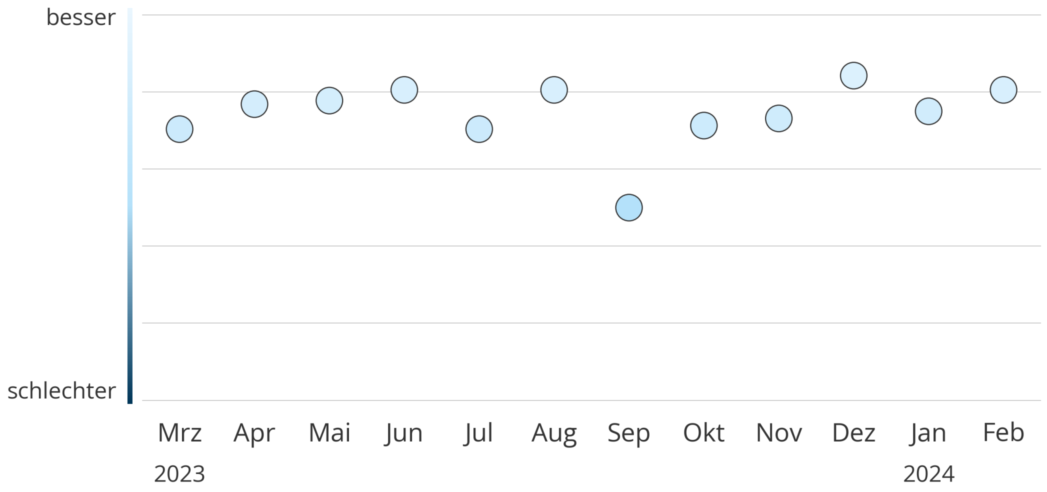

Soziale Funktion

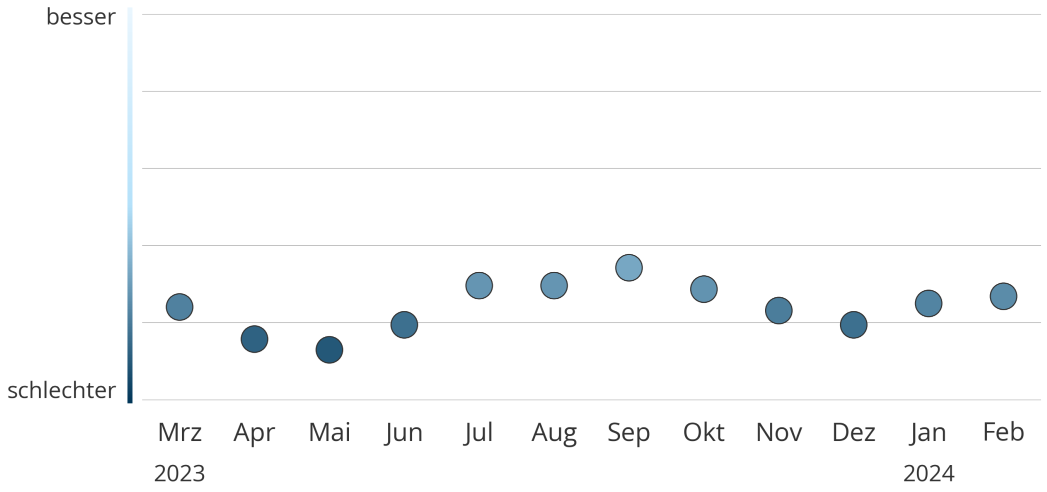

Diese Seite wurde bewusst leer gelassen.

Verschlechterungen Ihrer Befragungsergebnisse

Im Folgenden ist aufgelistet, wann in den letzten 12 Monaten der PRO B Studie eine Verschlechterung Ihrer Antworten in den Fragebögen gemessen wurde. Bei der gemessenen Verschlechterung war Ihr Behandlungsteam angehalten, Sie innerhalb von 48 Stunden diesbezüglich zu kontaktieren.

	Datum	Auslöser
**1**	28-03-2023	Körperliche Funktion, Rollenfunktion, Magen-Darm-Beschwerden
**2**	04-04-2023	Körperliche Funktion, Rollenfunktion, Magen-Darm-Beschwerden
**3**	16-05-2023	Körperliche Funktion, Emotionale Funktion, Soziale Funktion
**4**	30-05-2023	Soziale Funktion, Atemnot, Magen-Darm-Beschwerden
**5**	24-10-2023	Emotionale Funktion, Kognitive Funktion, Soziale Funktion, Atemnot, Magen-Darm-Beschwerden
**6**	19-12-2023	Atemnot
**7**	23-01-2024	Rollenfunktion, Emotionale Funktion, Kognitive Funktion, Soziale Funktion, Atemnot
