# Supplementary material for: Quantitative assessment of visual designs for communicating patient-reported outcomes in breast cancer care to patients
Source: J Patient Rep Outcomes. 2025 Dec 20;10:12. doi: 10.1186/s41687-025-00984-0 (PMC12830510; doi:10.1186/s41687-025-00984-0)

Studienzentrum

Patient-ID

Erläuterung zur Interpretation der Befragungsergebnisse

Auf den nächsten Seiten sehen Sie den Verlauf Ihrer Ergebnisse zu Lebensqualität & Funktionsfähigkeit und zu Ihren Symptomen. Auf der unteren Achse ist die Zeit in Monaten abgebildet. Sie haben alle 3 Monate einen Fragebogen beantwortet.

Auf der linken Achse sehen Sie einen Farbverlauf. Für Symptome entspricht ein helles Blau weniger Symptomen und ein dunkles Blau mehr Symptomen. Für Lebensqualität & Funktionsfähigkeit entspricht ein helles Blau besserer Funktionsfähigkeit und ein dunkles Blau schlechterer Funktionsfähigkeit.

Lebensqualität beschreibt Ihr subjektives Wohlbefinden im Alltag.

Körperliche Funktion beschreibt, wie gut Ihr Körper den Alltag bewältigen kann (z.B. einen längeren Spaziergang machen).

Rollenfunktion bezieht sich darauf, wie gut Sie sich in der Lage sehen, alltägliche Aufgaben zu erfüllen und an Freizeitaktivitäten teilzunehmen.

Emotionale Funktion bezieht sich darauf, wie stark Sie Sorgen oder Gefühle wie Niedergeschlagenheit oder Reizbarkeit wahrnehmen.

Kognitive Funktion bezieht sich auf Ihre Konzentrationsfähigkeit und Aufmerksamkeitsleistung sowie Ihr Erinnerungsvermögen.

Soziale Funktion beschreibt, wie Sie Ihre Beziehungen zu anderen Menschen wahrnehmen und wie aktiv Sie an gemeinsamen Unternehmungen in Ihrem sozialen Umfeld teilnehmen.

Diese Seite wurde bewusst leer gelassen.

Schmerz

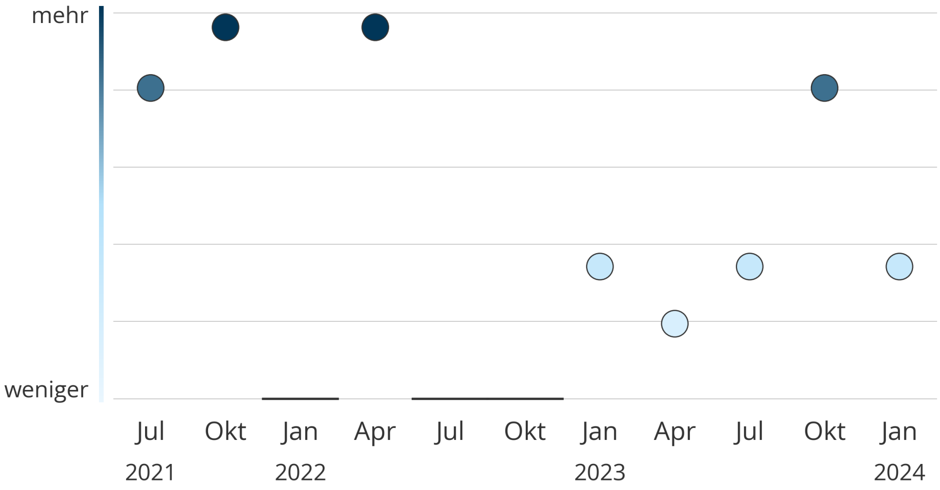

Atemnot

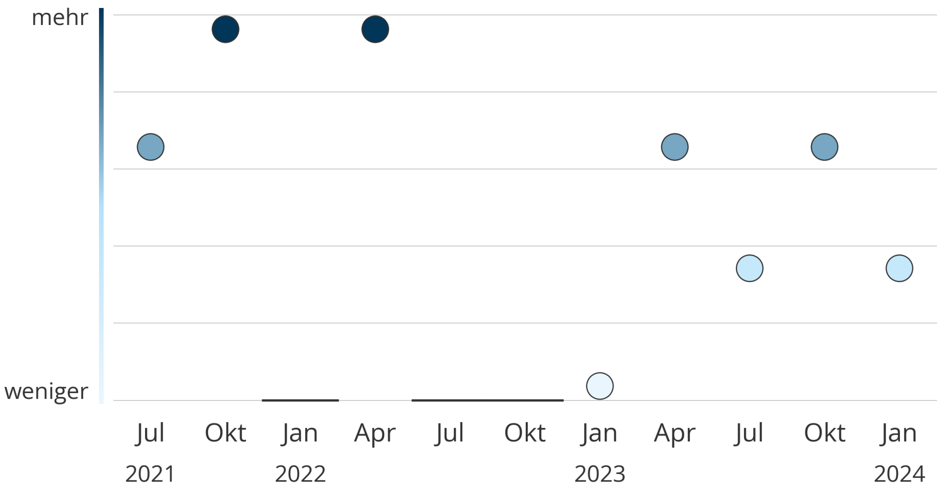

Fatigue (Erschöpfung)

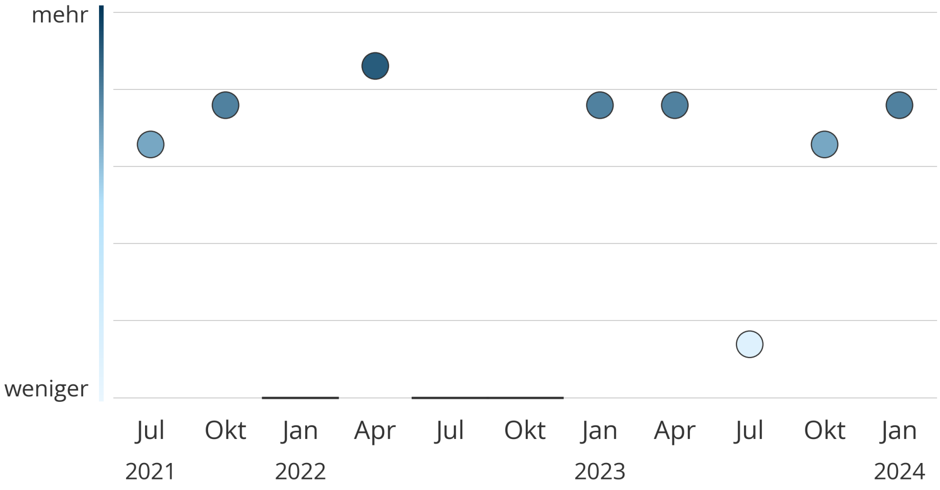

Übelkeit/Erbrechen

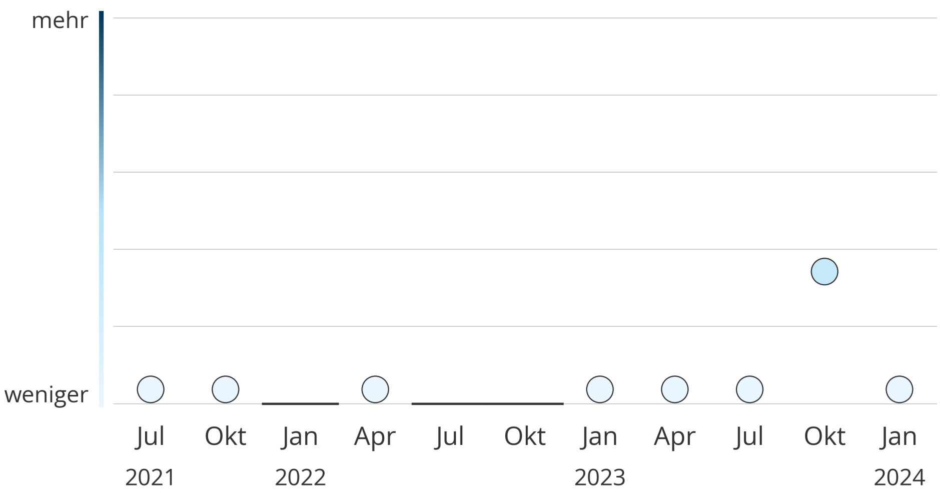

Verstopfung

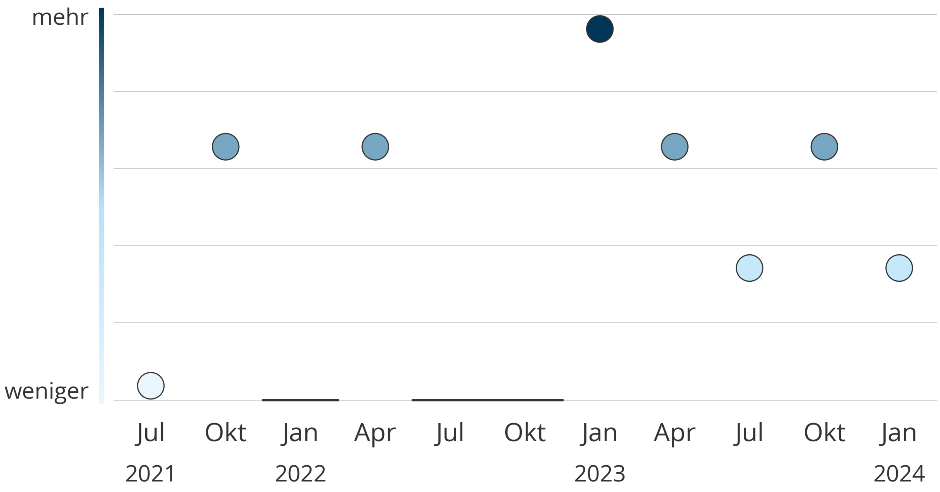

Durchfall

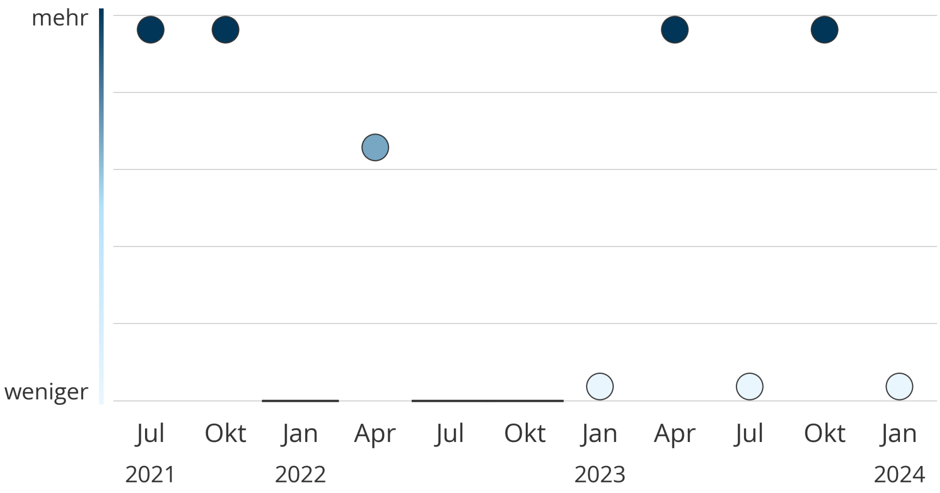

Appetitlosigkeit

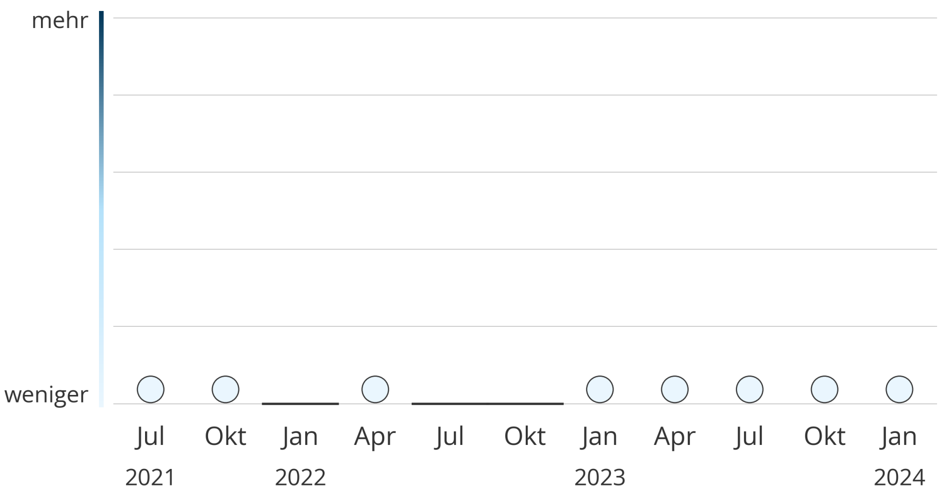

Schlaflosigkeit

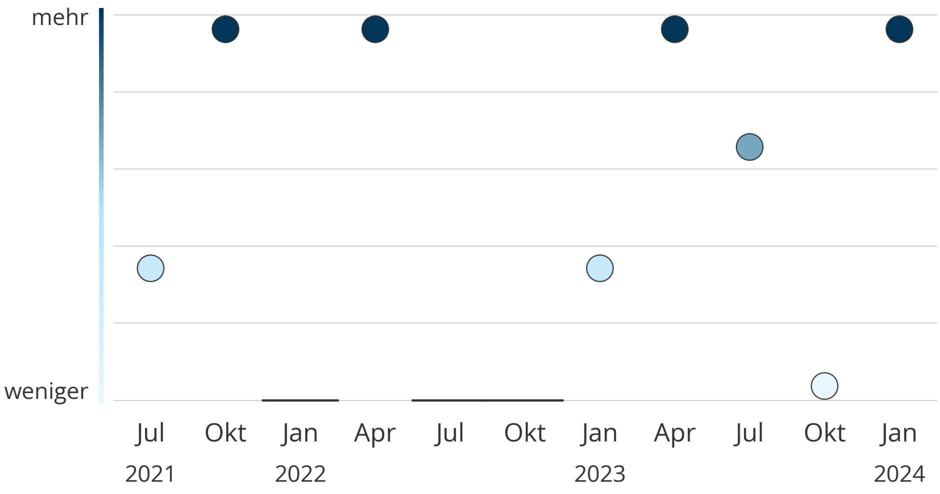

Finanzprobleme

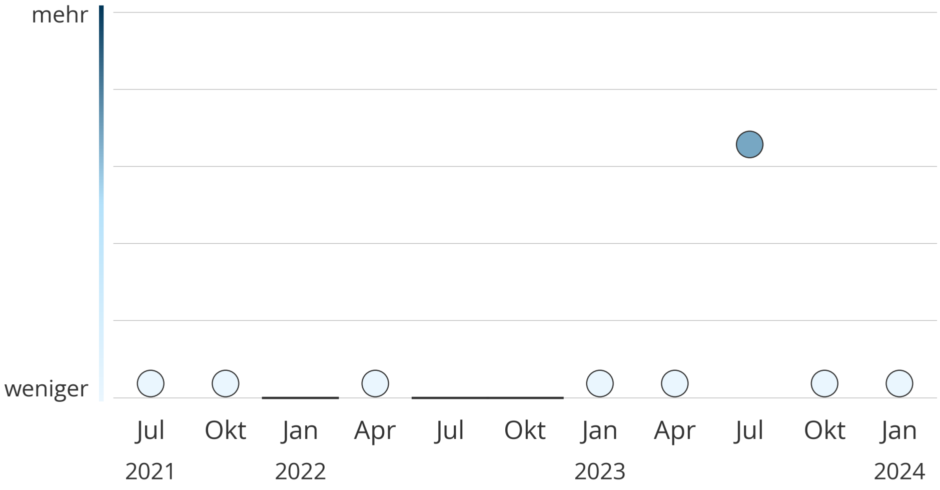

Lebensqualität

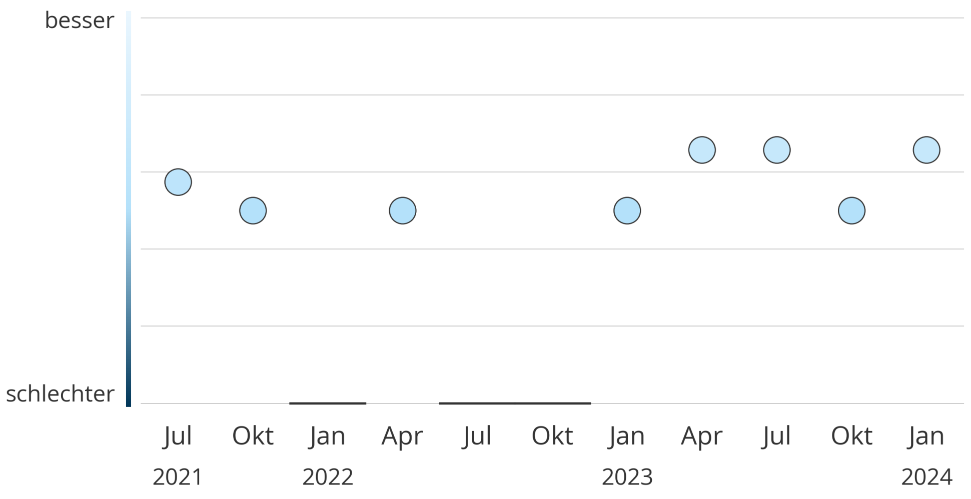

Körperliche Funktion

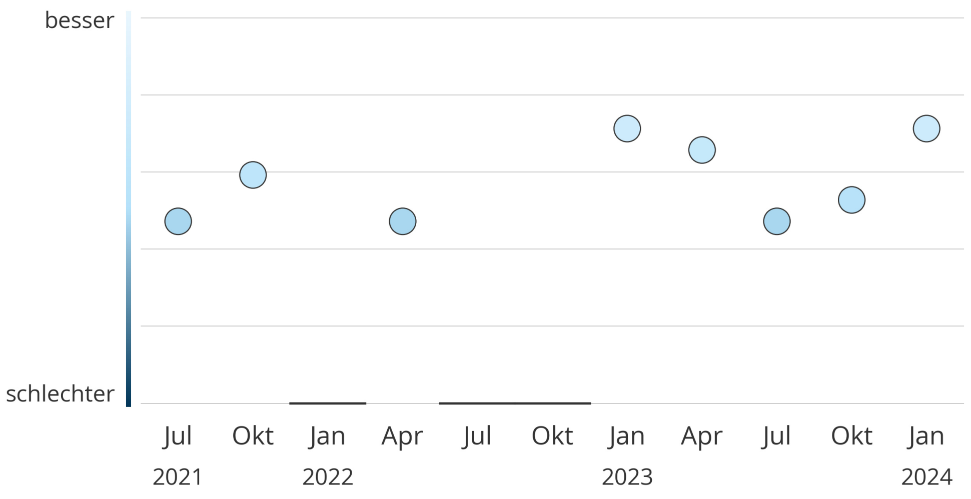

Rollenfunktion

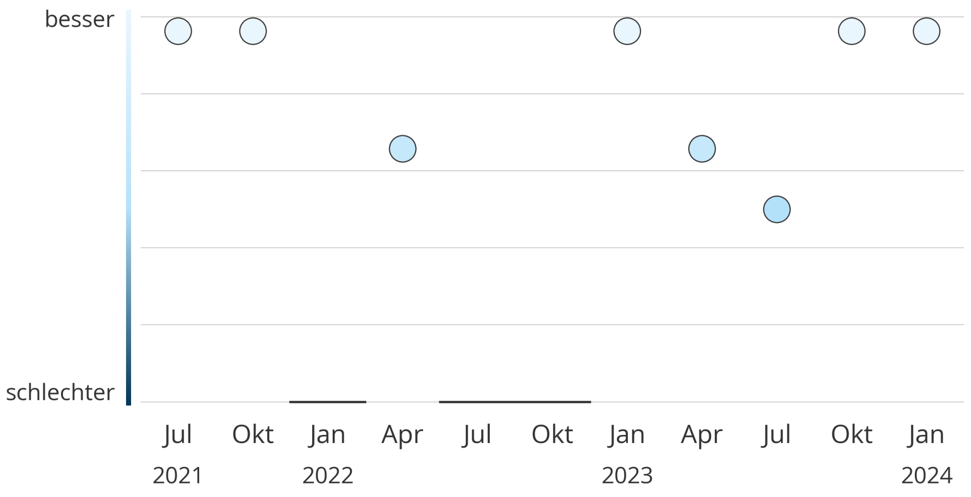

Emotionale Funktion

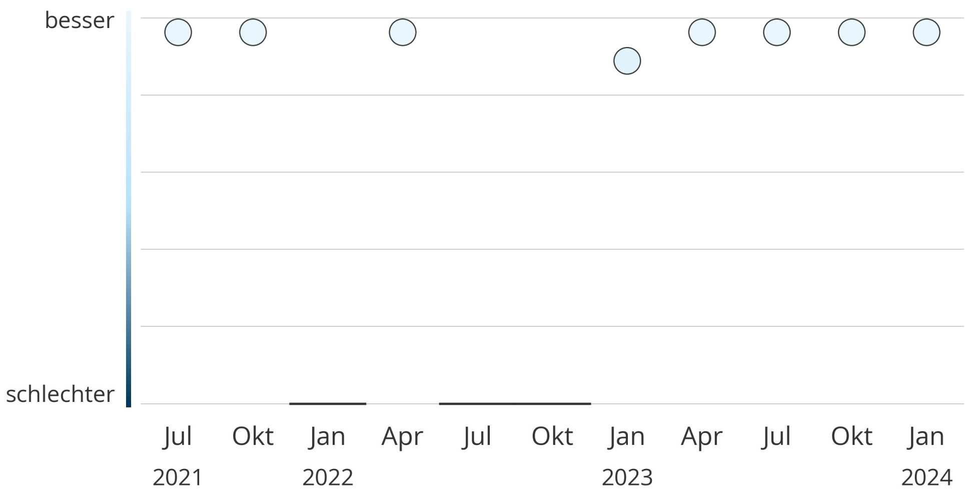

Kognitive Funktion

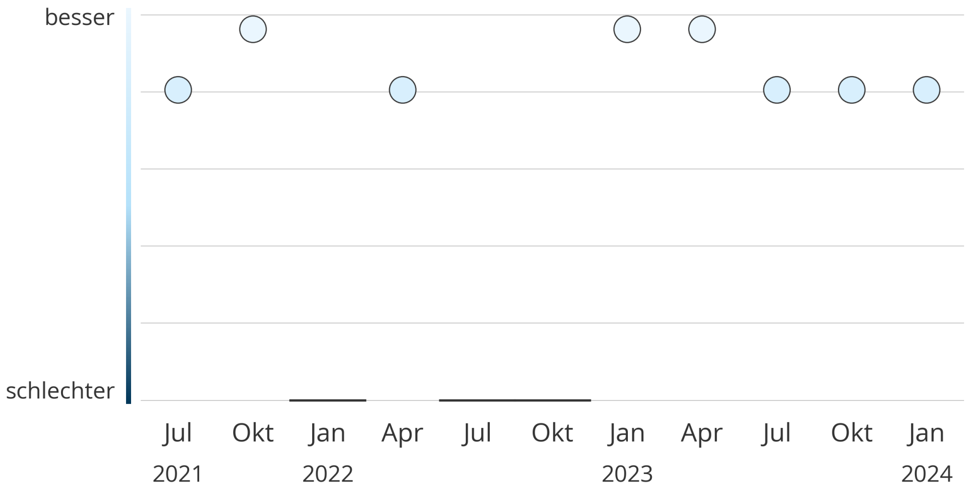

Soziale Funktion

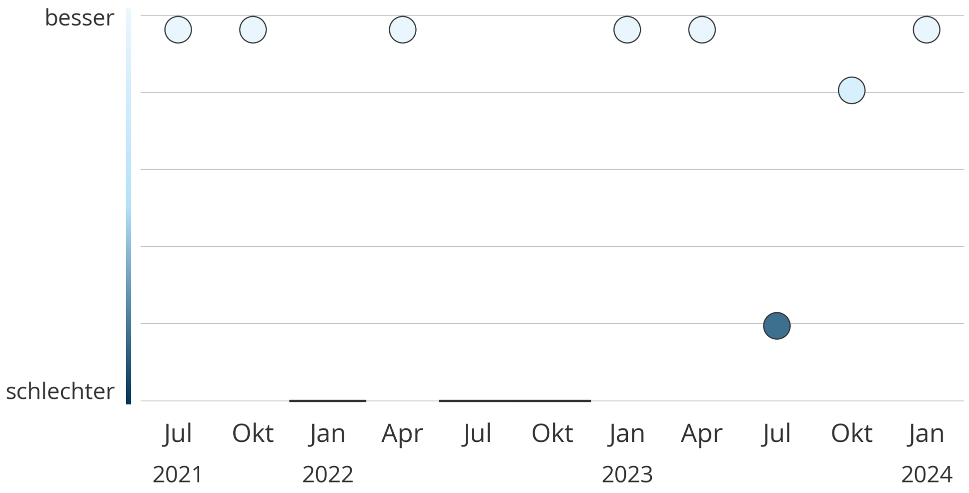

Supplement: Supplementary file 3 — Supplementary Material 3 [file 41687_2025_984_MOESM3_ESM.pdf]
