# Supplementary material for: Quantitative assessment of visual designs for communicating patient-reported outcomes in breast cancer care to patients
Source: J Patient Rep Outcomes. 2025 Dec 20;10:12. doi: 10.1186/s41687-025-00984-0 (PMC12830510; doi:10.1186/s41687-025-00984-0)
Supplement: Supplementary file 4 — Supplementary Material 4 [file 41687_2025_984_MOESM4_ESM.pdf]

Table 3 *Results of the evaluation survey on PRO visualizations assessing acceptance of visualization features, comprehensibility, clarity and usefulness.*

Item	Group[a]	Does not apply at all	Rather does not apply	Rather applies	Fully applies	No response	n with response	Sum Approval[b]	Percentage Approval[b]	Sum Rejection[c]	Percentage Rejection[c]	P-value
Understanding is easy	IG	0	3	44	102	3	149	146	96.1	3	2.0	0.776
	CG	1	3	36	82	2	122	118	95.2	4	3.2	
	**SUM**	**1**	**6**	**80**	**184**	**5**	**271**	264	**95.7**	**7**	**2.5**	
More guidance is needed	IG	98	37	5	10	2	150	15	9.9	135	88.8	0.987
	CG	83	21	7	12	1	123	19	15.3	104	83.9	
	**SUM**	**181**	**58**	**12**	**22**	**3**	**273**	**34**	**12.3**	**239**	**86.6**	
Labeling is understandable	IG	1	3	43	102	3	149	145	95.4	4	2.6	0.635
	CG	2	2	38	81	1	123	119	96.0	4	3.2	
	**SUM**	**3**	**5**	**81**	**183**	**4**	**272**	**264**	**95.7**	**8**	**2.9**	
Color gradient is helpful	IG	5	8	45	92	2	150	137	90.1	13	8.6	0.794
	CG	3	8	34	77	2	122	111	89.5	11	8.9	
	**SUM**	**8**	**16**	**79**	**169**	**4**	**272**	**248**	**89.9**	**24**	**8.7**	
Explanations of functional scales are necessary	IG	47	37	49	14	5	147	63	41.4	84	55.3	0.922
	CG	37	34	30	17	6	118	47	37.9	71	57.3	
	**SUM**	**84**	**71**	**79**	**31**	**11**	**265**	**110**	**39.9**	**155**	**56.2**	
"More" always at the top	IG	2	14	59	74	3	149	133	87.5	16	10.5	0.677
	CG	2	8	50	63	1	123	113	91.1	10	8.1	

	**SUM**	**4**	**22**	**109**	**137**	**4**	**272**	**246**	**89.1**	**26**	**9.4**	
Clinical information	IG	47	39	40	24	2	150	64	42.1	86	56.6	**<0.001**
	CG	20	29	33	41	1	123	74	59.7	49	39.5	
	**SUM**	**67**	**68**	**73**	**65**	**3**	**273**	**138**	**50.0**	**135**	**48.9**	
Reference to the general population	IG	65	28	31	26	2	150	57	37.5	93	61.2	0.165
	CG	39	36	21	27	1	123	48	38.7	75	60.5	
	**SUM**	**104**	**64**	**52**	**53**	**3**	**273**	**105**	**38.0**	**168**	**60.9**	
Reference to similar patients	IG	49	24	37	40	2	150	77	50.7	73	48.0	0.587
	CG	32	28	29	34	1	123	63	50.8	60	48.4	
	**SUM**	**81**	**52**	**66**	**74**	**3**	**273**	**140**	**50.7**	**133**	**48.2**	
Support for self-reflection	IG	3	15	54	72	8	144	126	82.9	18	11.8	0.100
	CG	6	10	59	47	2	122	106	85.5	16	12.9	
	**SUM**	**9**	**25**	**113**	**119**	**10**	**266**	**232**	**84.1**	**34**	**12.3**	
Improvement of communication	IG	8	25	63	49	7	145	112	73.7	33	21.7	0.079
	CG	7	26	60	27	4	120	87	70.2	33	26.6	
	**SUM**	**15**	**51**	**123**	**76**	**11**	**265**	**199**	**72.1**	**66**	**23.9**	
Strengthening of self-awareness	IG	7	13	58	67	7	145	125	82.2	20	13.2	0.061
	CG	6	11	65	39	3	121	104	83.9	17	13.7	
	**SUM**	**13**	**24**	**123**	**106**	**10**	**266**	**229**	**83.0**	**37**	**13.4**	

Recognition of support needs	IG<br>CG<br>**SUM**	9<br>6<br>**15**	23<br>11<br>**34**	59<br>65<br>**124**	54<br>39<br>**93**	7<br>3<br>**10**	145<br>121<br>**266**	113<br>104<br>**217**	74.3<br>83.9<br>**78.6**	32<br>17<br>**49**	21.1<br>13.7<br>**17.8**	0.985

Absolute counts and percentages are provided. Sum scores and p-values indicating significance are highlighted in bold.
[a] IG = intervention group; CG = control group
.[b] Approval: Aggregated responses for "Fully applies" and "Rather applies".
[c] Rejection: Aggregated responses for "Does not apply at all" and "Rather does not apply".
